# Exploring Hollandite-Type K*_y_* V*_x_* Ti_8–*x*_O_16_ (0.25 ≤ *x* ≤ 2) as Electrode Materials in Potassium-Ion Batteries (KIBs)

**DOI:** 10.1021/acs.inorgchem.4c05579

**Published:** 2025-04-22

**Authors:** Juan Andrés Nieto-Simón, Marta María González-Barrios, Adrián Gómez-Herrero, María Teresa Fernández-Díaz, Jesús Prado-Gonjal, Elizabeth Castillo-Martínez

**Affiliations:** † Departamento de Química Inorgánica, Facultad de Ciencias Químicas, Universidad Complutense de Madrid, Madrid E-28040, Spain; ‡ ICTS Centro Nacional de Microscopía Electrónica, Universidad Complutense de Madrid, Madrid E-28040, Spain; § Institut Laue Langevin, Grenoble F-38042, France

## Abstract

Hollandite-type oxides, K*_y_* V*_x_* Ti_8–*x*_O_16_, *x* = 0.25, 0.5, 0.75, 1, 1.25, 1.5, and 2, are synthesized via the citrate method and evaluated as potential electrode materials for potassium-ion batteries (KIBs). Neutron powder diffraction (NPD) confirms an undistorted *I*4/*m* structure, uniform K content (1.4 ≤ *y* ≤ 1.6), and high potassium isotropic displacement parameter (B_iso_). This decreases significantly for *x* ≥ 1, correlating with tunnel narrowing and vanadium’s stronger polarization. Transmission electron microscopy (TEM) techniques, including selected area electron diffraction (SAED), annular bright field (ABF), and high-angle annular dark-field scanning transmission electron microscopy (HAADF-STEM) identify superstructure reflections assigned to potassium/vacancy short-range order along the *c* axis with disorder between tunnels. Magnetic studies reveal paramagnetic behavior down to 2 K, with antiferromagnetic interactions at low temperature except for *x* = 0.25 composition, which exhibits ferromagnetic interactions. The experimental magnetic moment suggests a low Ti^3+^ content, with notable deviations at *x* = 1.25. The electrochemical performance is assessed via galvanostatic cycling using 2.5 M potassium bis­(fluorosulfonyl)­imide (KFSI) in triethyl phosphate (TEP) as electrolyte. At a rate of C/10, 2 K^+^ are reversibly de/inserted per formula unit, comparable to K_0.17_TiO_2_. At C/5, K_1.5_V_0.75_Ti_7.25_O_16_ demonstrates a reversible de/insertion of 1 K^+^/f.u., highlighting its potential for rechargeable KIBs.

## Graphical abstract

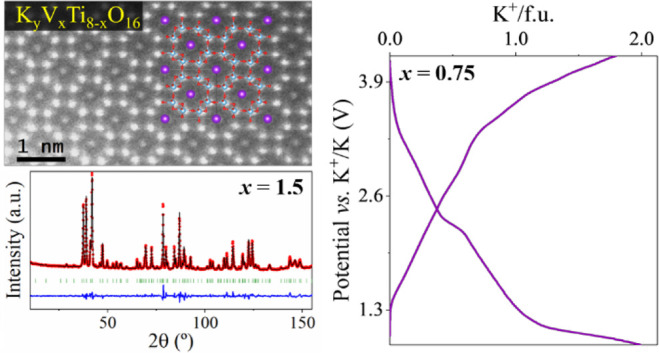

## Introduction

Nowadays, fossil fuels are being replaced with other energy sources due to the high CO_2_ emissions they produce, which lead to global warming and contribute negatively to the greenhouse effect.^1,2^ This has given rise to the need for research into renewable energies. However, many of these sources provide energy intermittently, making it necessary to couple them with storage systems that can store the excess energy produced at peak times, for later use when the source is unavailable.^3^ Among the different energy storage methods, electrochemical systems, such as batteries, fuel cells, and supercapacitors, stand out because of their high energy density, efficiency, and scalability.^3^ These systems convert and store energy through reversible chemical reactions, making them suitable for a wide range of applications from portable electronics to electric vehicles.

Within this category, rechargeable lithium-ion batteries (LIBs) are the leading storage technology, offering superior performance in terms of energy density, cycle life, and rechargeability. The operation of a LIB relies on a reversible redox reaction between the anode (reducing agent) and cathode (oxidizing agent), which are electronically connected via an external circuit and ionically linked through an electrolyte, typically LiPF_6_ dissolved in organic solvents like ethylene carbonate (EC) and dimethyl carbonate (DMC).^4,5^

However, although LIBs are currently dominating the battery market, the abundance of Li in the Earth’s crust is limited (0.0017%),^6^ and it is located in specific geographical areas.^7–9^ Due to these resource limitations, “post-Li-ion batteries” have been studied in recent years to replace LIBs, especially in large-scale applications. Currently, the interest in the development of Na^+^ and K^+^ insertion science is booming, due to the higher availability of these elements in the Earth’s crust (Na = 2.3%, K = 1.5%)^6^ and, therefore, the lower cost of their batteries compared to LIBs.^4,7^ Other properties such as the relatively low standard reduction potential of K (−2.936 V vs SHE), quite similar to that of Li (−3.040 V vs SHE),^10^ position KIBs as strong competitors to LIBs and Na-ion batteries (NIBs). Furthermore, KIBs can leverage a lower K^+^/K redox potential (−2.88 V) in certain organic electrolytes, such as propylene carbonate (PC),^10^ which can potentially expand the voltage window vs Li and enhance energy density. Another critical advantage is the weaker Lewis acidity of K-ion, which results in smaller solvated ion sizes,^11^ thereby improving ionic conductivity, transport efficiency, and diffusion of solvated K-ions across the electrolyte/electrode interface. Moreover, K does not alloy with Al at low voltages (similar to Na), allowing Al to replace Cu as the anode current collector, further reducing cell production costs.^11,12^

In terms of the positive active material, layered oxides,^13^ polyanionic compounds^14^ and Prussian blue analogues^15^ are part of commercial NIBs, while hard carbon is the preferred anode, due to the inability of bare Na^+^ to intercalate into graphite as Na plating occurs at higher voltages.^16^ In the field of KIBs, in 2004, Ali Eftekhari introduced the first KIB prototype using a Prussian blue cathode, demonstrating 500 cycles with a 12% capacity fade.^17^ A year later, a patent was filed for the use of potassium hexafluorophosphate (KPF_6_) as a potential electrolyte for KIBs.^18^ In 2007, Starsway Electronics, a Chinese company, launched the first potassium battery-powered portable media player.^19^ Despite early progress, KIB research decayed for years due to safety concerns and the dominance of LIB and SIB technologies. However, interest has resurged in the past decade. In 2024, Group1, a U.S. company, announced the commercialization of a KIB made with a K-Prussian White cathode and a graphite anode.^20^

One promising approach for these new batteries is the exploration of hollandite-type compounds as electrodes, which offer unique structural properties that could enhance ion storage and transport. Hollandite-type compounds have the chemical composition A*_x_* M_8_O_16_, where the A species corresponds to a mono or divalent cation (K^+^, NH_4_^+^, Ba^2+^, etc.) and M refers to a transition metal with mixed oxidation states (V, Ti, Mn, etc.).^21–25^ The M_8_O_16_ network, with two types of 1D tunnels, allows the insertion/extraction of cations and small molecules, such as water, into their wide channels as shown in Figure 1.

**Figure 1:**
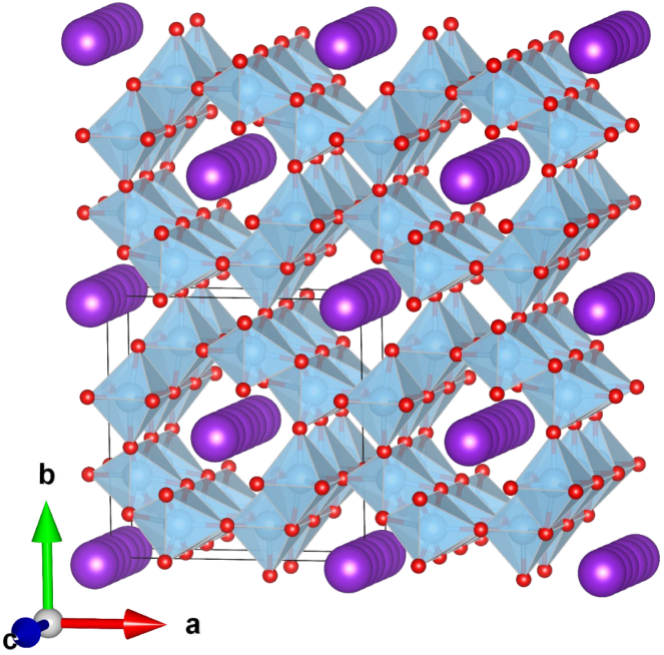
Schematic representation of the hollandite-type TiO_2_ structure in the *ab* plane, where TiO_6_ coordination octahedra are depicted in blue and tunnels of different sizes are shown. The smallest tunnels (1 × 1) contain no cations, while the largest tunnels (2 × 2) accommodate K^+^ (purple spheres) along the [001] direction.

The large tunnels can be occupied by A^n+^ cations up to a maximum of *y* = 2 in the case of K and up to 6 in the case of smaller Li.^26–28^ Large cations serve as templates for the hollandite structure. Moreover, charge transfer from the A cations in the tunnels to the M_8_O_16_ hollandite framework generates mixed oxidation states for the M species.^29^ Hosting a variable number of different cations and molecules in their tunnels allows hollandite-type materials to tune their physical and chemical properties, such as electrical, magnetic, or optical characteristics, for different applications.^26,30^

The aristotype hollandite belongs to the *I*4*/m* space group. However, sometimes, hollandite-type materials, such as Mn minerals, are slightly distorted and present lower symmetry. In 1982, Post et al.^31^ identified a monoclinic distortion (space group *I*2*/m*) if the *r*_M_/*r*_A_ ratio is higher than 0.48, where *r*_A_ is the average ionic radii of the tunnel cations and *r*_M_ is the average ionic radii of the octahedral cations. Cheary^32^ suggested that the combination of large-sized A-cations in the tunnels (e.g., K^+^ and Ba^2+^) and small-sized M-cations in the octahedra promotes the tetragonal phase, while smaller A-cations paired with larger M-cations favor the monoclinic structure. Years later, Zhang and Burnham^33^ proposed another predictive measure for determining the symmetry of hollandites. They suggested that if *r*_A_ > √2­(*r*_O_ + *r*_M_)–*r*_O_, where *r*_O_ is the average ionic radii of oxygens, the structure could not be monoclinic, while if *r*_A_ < √2­(*r*_O_ + r_M_)–*r*_O_–0.15 the material could not be tetragonal. This transition is thus influenced by the specific chemical composition of the material and the size and distribution of cations within the lattice and tunnels.^22,34^ Additional microstructural distortions have been observed in transmission electron microscopy in some instances, usually due to the ordering of the A-cations and their vacancies within the tunnels.^35–37^

Because of their tunneled structure, hollandite-type materials have been studied as electrodes for rechargeable LIBs, NIBs, and KIBs. On the one hand, regarding negative electrode materials, K_0.008_TiO_2_ (or K_0.064_Ti_8_O_16_, for a consistent nomenclature) can act as relatively high voltage (2 V vs Li^+^/Li) anode material for LIBs, with increasing reversible capacity as the K content in the tunnel decreases.^38^ Up to 4 Li^+^/f.u. can be reversibly de/inserted at high rates of 5 C. Doping on the Ti sites has been attempted without successful electrochemical performance in the case of Al^39^ or Mn^40^ doping of K*_y_* Ti_1–*x*_M*_x_* O_2_. Nb substituted K_0.19_Ti_0.9_Nb_0.1_O_2_ and K_0.06_Ti_0.9_Nb_0.1_O_2_ demonstrated promising performance as rechargeable electrode materials, with Li insertion capacity increasing significantly as K content decreased.^41^ X-ray absorption spectroscopy revealed that both Ti and Nb participated in the redox reactions during the charge–discharge process, with Nb exhibiting greater redox flexibility than Ti within the voltage range of 1.0–3.0 V. An initial insertion capacity of 130 mA·h·g^–1^ was achieved by maximizing K^+^ extraction while preserving the tunnel structure. Furthermore, hollandite-structured TiO_2_ was also evaluated as an anode material for NIBs,^42^ exhibiting a reversible capacity of 85 mA·h·g^–1^ (2.03 Na^+^/f.u.) in the potential range of 0.2–2.5 V vs Na^+^/Na.

On the other hand, regarding positive electrode materials, α-MnO_2_ and analogues have been studied as cathode materials in several chemistries, including LIBs^24,25,43,44^ which demonstrated a reversible specific capacity of 165 mA·h·g^–1^ after 20 cycles.^44^ Furthermore, this hollandite-type material is also a potential positive electrode of rechargeable Mg-ion batteries.^45–47^ For NIBs, nanosized hollandite-type Na*_x_* VO_1.75_(OH)_0.5_ ^48^ exhibited a high specific capacity of approximately 351 mA·h·g^–1^ at C/20 within a voltage range of 1.0–3.7 V (vs Na^+^/Na) and 70% capacity retention after 200 cycles. The open tunnel structure and nanoscale particle size seemed to contribute to its excellent power capability, maintaining 56% of the initial capacity at a high rate of 7 C compared to that measured at C/20. In the case of K^+^ insertion and extraction, given the larger size of K^+^ vs Li^+^ or Na^+^, only the large tunnels can be occupied and less cations/f.u. can be accommodated, nonetheless acidic K^+^ leaching seems unnecessary. For KIBs, hollandite-type K_0.17_TiO_2_ ^49^ exhibited a reversible capacity of 70 mA·h·g^–1^ (1.81 K^+^/f.u.) at C/10 in a potential range of 1.0 to 4.0 V, with a capacity retention of 94% after 300 cycles at C/20.

Given the good capacity retention of K_0.17_TiO_2_ in KIBs, we have synthesized hollandite-type materials in this work, introducing V at the Ti site to potentially modify their electronic properties and study its influence on their electrochemical performance for KIBs. Nominal compositions K*_y_* V*_x_* Ti_8–*x*_O_16_ (0.25 ≤ *x* ≤ 2) have been synthesized for the first time by using a citrate method. Their crystal structure was characterized by powder X-ray diffraction (PXRD) and neutron powder diffraction (NPD). TEM analysis reveals that up to *x* = 1.5, samples show superstructure reflections, which are assigned to K/vacancy ordering. The electrochemical performance of hollandite-type K*_y_* V*_x_* Ti_8–*x*_O_16_ materials as electrodes for K-ion batteries (KIBs) is evaluated through galvanostatic charge–discharge measurements. Using K_2_V_2_Ti_6_O_16_ as a starting composition, parameters such as the electrolyte and potential window are optimized. Optimization of the electrolyte showed that 2.5 M KFSI in TEP provided higher reversible capacities and stability up to 4.2 V compared to 3.9 M KFSI in DME. Among the studied compositions, K_1.5_V_0.75_Ti_7.25_O_16_ demonstrated the best performance, with a reversible capacity of 80 mA·h·g^–1^ (2 K^+^/f.u.) in cycle 2, gradually decreasing to 58 mA·h·g^–1^ (1.45 K^+^/f.u.) by cycle 5. Moreover, a maximum of 1 K^+^/f.u. at C/5 for this nominal composition can be reversibly extracted.

## Experimental Procedure

### Synthesis

Vanadium–titanium hollandites with nominal compositions of K_1.5_V_0.25_Ti_7.75_O_16_, K_1.5_V_0.5_Ti_7.5_O_16_, K_1.5_V_0.75_Ti_7.25_O_16_, K_1.5_VTi_7_O_16_, K_1.5_V_1.25_Ti_6.75_O_16_, K_1.5_V_1.5_Ti_6.5_O_16_, K_1.75_V_1.75_Ti_6.25_O_16_, and K_2_V_2_Ti_6_O_16_ were synthesized using the citrate method. For the synthesis of K*_y_* V*_x_* Ti_8–*x*_O_16_ (1.5 ≤ *x* ≤ 2), stoichiometric amounts of titanium­(IV) isopropoxide (C_12_H_28_O_4_Ti, 98%, Sigma-Aldrich), vanadium­(III) oxide (V_2_O_3_, 98%, Sigma-Aldrich), and potassium hydroxide (KOH, 84%, Merck) were used to obtain 2 g of the desired materials with composition *y* = *x*. However, to synthesize K*_y_* V*_x_* Ti_8–*x*_O_16_ (0.25 ≤ *x* ≤ 1.25), the amount of KOH used was calculated based on the stoichiometry K_1.5_V*_x_* Ti_8–*x*_O_16_. The first step of the synthesis involved mixing the reagents with a solution of citric acid (C_6_H_8_O_7_, 99%, Sigma-Aldrich). This solution was prepared in a 2:1 ratio with respect to the total moles of the reagents and dissolved in 30 mL of distilled water. Then, vanadium­(III) oxide and titanium­(IV) isopropoxide were introduced into a porcelain capsule and placed on a hot plate. The mixture was heated under stirring. Next, KOH (dissolved in *ca.* 30 mL of distilled water) was added to form a white putty, and distilled water (120 mL) was poured over the entire surface of the mixture. When the plate reached a temperature of 40–50 °C, a citric acid solution was added. The temperature was increased to 90–100 °C, and the reaction was maintained for 2 h until the mixture became dark and gel-like in consistency. After this, the gel was aged in an oven for 20 h at 80 °C. Then, calcination of the gel was conducted in a furnace at 600 °C in air for 12 h. To finalize the synthesis, the resulting powder was ground and compacted into a pellet. The pellet was then placed in a tubular furnace, where it was heated at 960 °C in the presence of forming gas (5% H_2_/95% N_2_) for 20 h and subsequently cooled to room temperature.

### Structural Characterization

Powder X-ray diffraction (PXRD) measurements were performed in a multipurpose diffractometer PANalytical model X’Pert Pro MPD in the 2θ range from 10° to 90° with a step size of 0.033°, using CuKα radiation as the excitation source, with wavelengths λ_1_ = 1.540598 Å and λ_2_ = 1.544426 Å. Room temperature neutron powder diffraction (NPD) patterns were collected at the Institut Laue Langevin (ILL) in Grenoble, France, using a high-resolution two-axis diffractometer (D2B), with a wavelength λ = 1.594 Å, within the 2θ range from 5° to 160° and a step size of 0.05°. A measurement time of 3 h was required for each sample. This technique is therefore useful to differentiate V (Coh b = −0.3824)^50^ from Ti (Coh b = −3.438)^50^ of the synthesized compositions, which are indistinguishable in PXRD as they have a very similar Z. Vanadium is practically transparent to neutrons, deducing its content by difference with that of titanium. Furthermore, this technique makes it possible to determine Ti/V ratio and the O content (Coh b = 5.803)^50^ present in the sample, unlike PXRD, and K content (Coh b = 3.67).^50^ The resulting NPD data were analyzed using the Rietveld method with the FullProf refinement software.^51^ The chemical composition of K*_y_* V*_x_* Ti_8–*x*_O_16_ samples was also investigated using X-ray fluorescence (XRF; PANalytical wavelength dispersive spectrometer model Axios, 4 kW). Inductively coupled plasma-optical emission spectroscopy (ICP-OES) measurements were performed in a SPECTRO Arcos, optical emission spectrometer with ICP excitation source. Sample for analysis was prepared by digesting *ca.* 40 mg of finely ground powder in aqua regia (HNO_3_: 3 HCl) with a few drops of hydrofluoric acid. Three replicates were measured to study reproducibility and obtain an average compositional value (Table S2). Scanning electron microscopy (SEM) images were acquired on a JEOL 6400 JSM. This microscope features a semiquantitative energy-dispersive X-ray spectroscopy (EDS) microanalysis system that enables analysis with a resolution of 133 eV. All samples were previously coated with gold. ImageJ software was employed to determine the size of individual particles. A JEOL JEM 2100 HT microscope was used for selected area electron diffraction (SAED), convergent beam electron diffraction (CBED), and high-resolution transmission electron microscopy (HR-TEM). Gatan Digital Micrograph software was employed to analyze the micrographs obtained by HR-TEM, as well as the SAED and CBED images. High-angle annular dark-field (HAADF) and annular bright field (ABF) scanning transmission electron microscopy (STEM) images were obtained on a probe aberration-corrected JEOL ARM 200 cF microscope operated at 200 kV. The grids used in TEM and HAADF-STEM were prepared in air for pristine hollandite-type materials and under inert conditions (inside a glovebox) for oxidized and reduced materials. These grids were prepared with one drop of a suspensions of the pristine hollandite-type materials that had been dispersed previously in butanol and one drop of the suspensions of oxidized and reduced materials that had been dispersed previously in TEP.

### Magnetic Characterization

Magnetic properties of the as-prepared samples were measured using an MPMS-SQUID, Quantum Design. The temperature dependence of the magnetization was analyzed using the following procedure: first, the samples were cooled from 300 to 2 K without applying a magnetic field. Then, a magnetic field was applied, and the temperature-dependent magnetization was measured with an applied magnetic field H = 1000 Oe [zero field cooled (ZFC)] while increasing the temperature. Subsequently, the samples were cooled again with a constant applied magnetic field of 1000 Oe. Finally, the magnetization of the field cooled (FC) sample was recorded with an applied magnetic field H = 1000 Oe. The paramagnetic susceptibility data were fitted using the Curie–Weiss law (eq 1).

1 $$\chi_{T}=\chi_{D}+\frac{C}{T-\theta }$$

where *χ*_T_ is the total magnetic susceptibility (emu·mol^–1^·Oe^1–^), *χ*_D_ is the diamagnetic susceptibility (emu·mol^–1^·Oe^1–^), *C* is the Curie constant (emu·K·mol^–1^·Oe^1–^), *T* is the temperature (K), and θ is the Weiss constant (K). The diamagnetic contribution to the susceptibility was calculated according to Pascal’s constants.^52^

If eq 1 is linearized, the expression of eq 2 is obtained:

2 $$\frac{1}{\chi_{T}-\chi_{D}}=T\cdot \frac{1}{C}-\frac{\theta }{C}$$

where (*χ*_T_–*χ*_D_)^−1^ (mol·Oe·emu^–1^) is the *y* variable of the linear equation, *T* (K) is the *x* variable, *C* ^–1^ (mol·Oe·emu^–1^·K^–1^) is the slope, and −*θ·C* ^–1^ (mol·Oe·emu^–1^) is the ordinate at the origin.

### Electrochemical Measurements

The compound (powder) was mixed with C_65_ conductive carbon in a proportion of 70% active material and 30% carbon C_65_. The mixture was then placed under vacuum for approximately 20 h at 100 °C prior to bringing into the glovebox. The cells used for the electrochemical measurements were two-electrode stainless steel Swagelok-type cells. The cell components were washed with water and ethanol and then dried in an oven for at least 24 h at 80 °C. Afterward, both the components and the mixed powder were placed into a glovebox, with an argon atmosphere (O_2_ < 1 ppm and H_2_O < 1 ppm). To assemble the complete half-cell, first, a semicell was assembled in air, i.e., a plunger with its respective front and rear ferrules was inserted into the body, which already had a Kapton film inside, to avoid possible short circuits of the cells, and it was secured with a nut. Next, once this half-cell was put inside the glovebox, the mixture of active material and C_65_ was deposited inside the body on the plunger of this half-cell. A 13 mm diameter circular glass fiber sheet (Whatman) was then added to function as a separator between the two electrodes. Later, electrolyte was added to this glass fiber. Regarding potassium metal, this was flattened with a roller until a thin film was obtained. The oxidation on the surface of this film was removed by using a brush and 0.1 mL of hexane on both sides of the thin film until the metal exhibited a bright silver color. Then, this film was cut into disks by employing an 11 mm diameter circular die cutter. Finally, the disk of metallic potassium was deposited on top of the glass fiber soaked with the electrolyte. Once this was done, the half-cell was completed with another plunger and its respective ferrules and tightened with another nut. To conclude, the nuts were tightened with spanners to make everything secure. To conduct an electrochemical study of the synthesized materials, galvanostatic cycling of the materials was performed. The electrochemical performance of these materials was studied during 25 galvanostatic charge–discharge cycles at different C rates (C/10–C/5–C/2–C–C/10) performing 5 cycles in each C rate. The cycling tests started with K^+^ extraction, meaning the material was initially oxidized. The theoretical capacity of each sample was calculated as a function of 2 K^+^/f.u., since 2 is the maximum number of K^+^ that can be inserted into the tunnels of the hollandite structure.^26^ The potential window analyzed was 0.9–4.2 V, and the electrolytes evaluated were 3.9 M KFSI in dimethoxyethane (DME) and 2.5 M KFSI triethylphosphate (TEP). The equipment used to conduct the electrochemical measurements was an Arbin model BT2143 multichannel potentiostat-galvanostat, using its specific software to establish the appropriate measurement conditions for the cells.

## Results and Discussion

### Powder X-ray Diffraction

Figure 2a presents PXRD patterns of the eight prepared black powdered K*_y_* V*_x_* Ti_8–*x*_O_16_ (0.25 ≤ *x* ≤ 2) samples, each with varying nominal V content. All patterns display the characteristic Bragg reflections of a tetragonal *I*4*/m* (no. 87) hollandite-type structure, with no noticeable differences between them. The tetragonal structure is in good agreement with the expected symmetry for *r*_M_/*r*_A_ < 0.48,^31^ calculated considering the average radii for the NPD Ti/V-derived content. This ratio *r*_M_/*r*_A_ varies from 0.40 to 0.42 for all synthesized compositions. As it can be observed in Figure 2b, there is a progressive shift to higher angles in the reflections as the vanadium content *x* increases. This shift is indicative of lattice parameter contraction confirming the compositional diversity of the synthesized samples.

**Figure 2:**
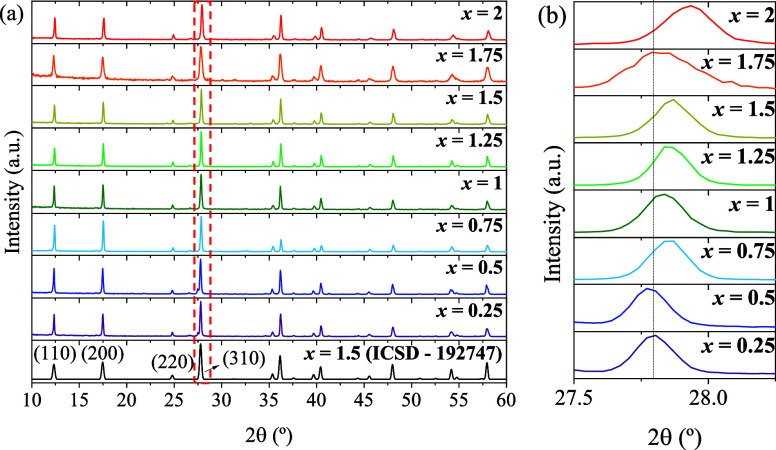
(a) Comparison between PXRD patterns of the as-prepared K_*y*_V_*x*_Ti_8–*x*_O_16_ (0.25 ≤ *x* ≤ 2) and reference K_1.5_V_1.5_Ti_6.5_O_16_.^53^ (b) Enlargement of (310) reflection observed in (a).

An irregularity in the shift is observed for the *x* = 1.75 nominal composition, accompanied by a slightly broader (310) reflection (Figure 2b). Nonetheless, no lowering of symmetry was found by electron microscopy CBED (see Figure 5c). The K nominal content *y* = 1.5 was chosen for samples with *x* ≤ 1.5, as a lower potassium content led to impurities, while samples with *x* ≥ 1.5 and *y* ≥ 1.5 resulted in effective K contents close to 1.5.

### Powder Neutron Diffraction

Room temperature NPD patterns for as-prepared K*_y_* V*_x_* Ti_8–*x*_O_16_ (0.25 ≤ *x* ≤ 2) samples were collected, as shown in Figure 3a. The structure of these compounds was confirmed from the Rietveld refinement of the neutron diffraction data in the tetragonal *I*4*/m* (#87) space group. Figure 3b shows the refined crystal structure of *x* = 2 nominal composition as a representative of hollandite-type K*_y_* V*_x_* Ti_8–*x*_O_16_, where (V/Ti)­O_6_ coordination octahedra are represented in blue, while the oxygen atoms are shown in red. Refinement of these NPD data discards a long range V/Ti ordering in the hollandite-type structure of these materials; there is only a partial occupancy of the single *8h* Wyckoff position by both atoms. There are two crystallographically distinct oxygen atoms, O1 and O2, with the (V/Ti)­O_6_ octahedra being distorted. Taking the channels created along the *c* axis as a reference, O1 atoms are located at the center of the edges of the square section formed by these tunnels, while the O2 atoms are positioned at the vertices. Furthermore, refinement of the NPD data provides evidence of partial K occupancy at the *2b* and *4e* Wyckoff positions.

**Figure 3:**
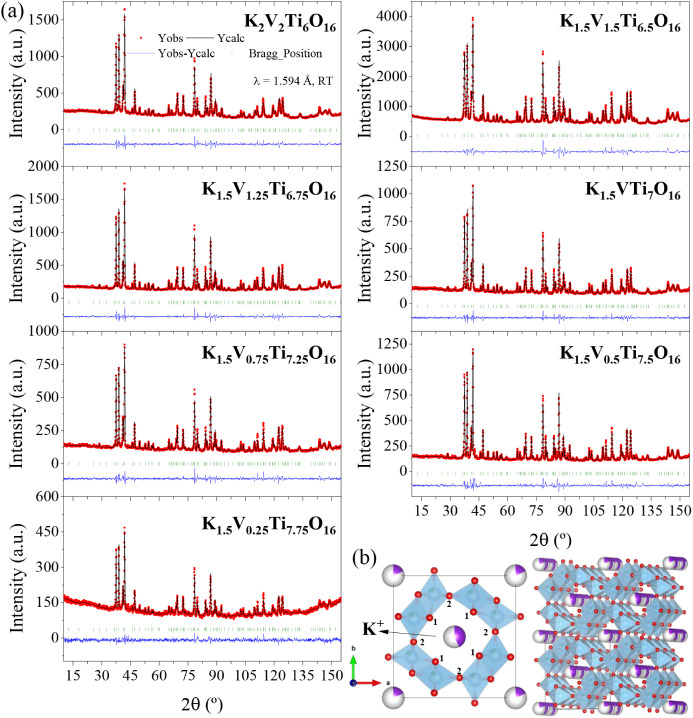
(a) Observed (red dots), calculated (black, full line), and difference (blue line) neutron powder diffraction profile for refined nominal compositions: K_2_V_2_Ti_6_O_16_, K_1.5_V_1.5_Ti_6.5_O_16_, K_1.5_V_1.25_Ti_6.75_O_16_, K_1.5_VTi_7_O_16_, K_1.5_V_0.75_Ti_7.25_O_16_, K_1.5_V_0.5_Ti_7.5_O_16_, and K_1.5_V_0.25_Ti_7.75_O_16_. The vertical green bars correspond to the allowed Bragg reflections. (b) Hollandite-type structure representation obtained from the Rietveld refinement data.

The structural parameters obtained from NPD data for all V/Ti compositions after Rietveld refinement are listed in Table 1, along with agreement factors (*R* values). In all cases, good fits were achieved with a single hollandite tetragonal phase, with *R* values below 5%, except for the *x* = 0.25 sample, where statistics are lower due to a smaller amount of sample available.

**Table 1:** Refined Structural Parameters Obtained from NPD Data for the Following Nominal Compositions: K_1.5_V_0.25_Ti_7.75_O_16_, K_1.5_V_0.5_Ti_7.5_O_16_, K_1.5_V_0.75_Ti_7.25_O_16_, K_1.5_VTi_7_O_16_, K_1.5_V_1.25_Ti_6.75_O_16_, K_1.5_V_1.5_Ti_6.5_O_16_, and K_2_V_2_Ti_6_O_16_ at Room Temperature

K*_y_* V*_x_* Ti_8–*x*_O_16_		*x* = 0.25	*x* = 0.5	*x* = 0.75	*x* = 1	*x* = 1.25	*x* = 1.5	*x* = 2
**Temperature (K)**		298.15	298.15	298.15	298.15	298.15	298.15	298.15
**Space group**		*I*4*/m*	*I*4*/m*	*I*4*/m*	*I*4*/m*	*I*4*/m*	*I*4*/m*	*I*4*/m*
*a* **=** *b* **(Å)**		10.1573(6)	10.1610(5)	10.1565(4)	10.1488(3)	10.1509(4)	10.1530(5)	10.1370(5)
*c* **(Å)**		2.963(2)	2.9620(2)	2.9626(2)	2.9638(1)	2.9650(2)	2.9651(2)	2.9659(2)
**Volume (Å** ^**3**^ **)**		305.65(3)	305.81(3)	305.61(3)	305.26(2)	305.51(2)	305.65(3)	304.77(3)
Ti**1**/V**1** (*8h*) (***x*** ***y*** 0)	*x*	0.351(1)	0.351(1)	0.351(1)	0.3493(8)	0.3500(9)	0.351(1)	0.350(1)
	*y*	0.167(2)	0.168(1)	0.167(1)	0.166(1)	0.167(1)	0.167(1)	0.166(1)
	B_iso_ (Å^2^)	0.6(3)	0.9(2)	0.8(2)	0.9(2)	0.8(2)	0.6(2)	0.7(3)
	SOF Ti1	0.960(7)	0.98(1)	0.91(1)	0.90(1)	0.90(1)	0.841(7)	0.841(9)
	SOF V1	0.040(7)	0.02(1)	0.09(1)	0.10(1)	0.10(1)	0.159(7)	0.159(9)
**K1 (***2b***) (0 0**1/2)	B_iso_ (Å^2^)	3(2)	4(3)	3(2)	1(1)	1(1)	1(1)	1(1)
	SOF	0.44(4)	0.47(5)	0.45(4)	0.39(2)	0.37(2)	0.34(1)	0.43(1)
**K2 (** *4e* **) (0 0 z)**	*z*	0.75(6)	0.8(1)	0.78(4)	0.75(2)	0.79(3)	0.78(2)	0.80(3)
	B_iso_ (Å^2^)	3(2)	4(3)	3(2)	1(1)	1(1)	1(1)	1(1)
	SOF	0.18(4)	0.10(5)	0.16(4)	0.16(2)	0.17(2)	0.20(1)	0.18(1)
**O1 (***8h*) (***x*** ***y*** 0)	*x*	0.156(1)	0.1557(9)	0.1561(8)	0.1561(6)	0.1554(6)	0.1556(7)	0.1555(8)
	*y*	0.2033(7)	0.2049(6)	0.2047(5)	0.2043(4)	0.2041(4)	0.2040(5)	0.2030(5)
	B_iso_ (Å^2^)	0.4(1)	0.5(1)	0.5(1)	0.5(1)	0.5(1)	0.48(7)	0.3(1)
	SOF	1	1	1	1	1	1	1
**O2 (***8h*) (***x*** ***y*** 0)	*x*	0.5412(8)	0.5400(6)	0.5402(6)	0.5409(4)	0.5402(5)	0.5405(5)	0.5407(6)
	*y*	0.164(2)	0.165(1)	0.165(1)	0.1659(8)	0.1666(8)	0.165(1)	0.166(1)
	B_iso_ (Å^2^)	0.4(1)	0.5(1)	0.5(1)	0.5(1)	0.5(1)	0.48(7)	0.3(1)
	SOF	1	1	1	1	1	1	1
***R* factors**								
***R***_**p**_		3.01	3.91	3.51	3.47	3.69	2.90	2.95
***R***_**wp**_		3.91	5.63	4.87	4.62	5.12	4.07	4.14
**R_exp_**		1.31	1.18	1.28	1.26	1.08	1.63	0.95
***R***_**Bragg**_		9.67	5.98	5.39	3.08	4.01	4.79	3.98

Table 1 shows a minor change in the lattice parameter vs composition, with a tendency for the *a* lattice parameter to decrease and *c* lattice parameter to increase as vanadium replaces titanium, more noticeably at the end composition (*x* = 2). The variation of *a* lattice parameter is directly correlated with the variation in cell volume, which in overall decreases as the smaller V^3+^ (*r* = 0.64 Å)^54^ replaces Ti^3+^ (*r* = 0.67 Å).^54^ This correlation occurs because, although the *c* lattice parameter slightly increases, this variation is minimal; i.e., it remains almost constant (≈2.9639 Å) throughout the compositional range. The *a* lattice parameter decreases, causing the tunnel to become narrower as vanadium replaces titanium.

Looking at the refined interatomic distances (Figure 4a), the longest interatomic distance in the octahedra, Ti–O1_ax_ (blue), follows a trend similar to that of the longest Ti–O2_eq_ distances (purple) of the octahedra, while the shortest Ti–O1_eq_ distance (red) shows a trend similar to that of the shortest Ti–O2_ax_ distance (orange). In general, the increase in V content leads to less distorted octahedra. Figure 4b exhibits the trend in Ti–O2–Ti angles, where the largest Ti–O2–Ti angle along the *b* axis for the K_1.5_V_0.5_Ti_7.5_O_16_ nominal composition corresponds to the smallest Ti–O2–Ti angle along the *c* axis for that composition, showing a correlation between them as well as with the interatomic distances. While the trends between Ti–O2–Ti angles along *c* and *b* axes are opposite, the trend observed between Ti–O1–Ti angles along the *c* axis and the unit cell diagonal is similar (as labeled in a (V/Ti)­O_6_ octahedra in Figure 4c).

**Figure 4:**
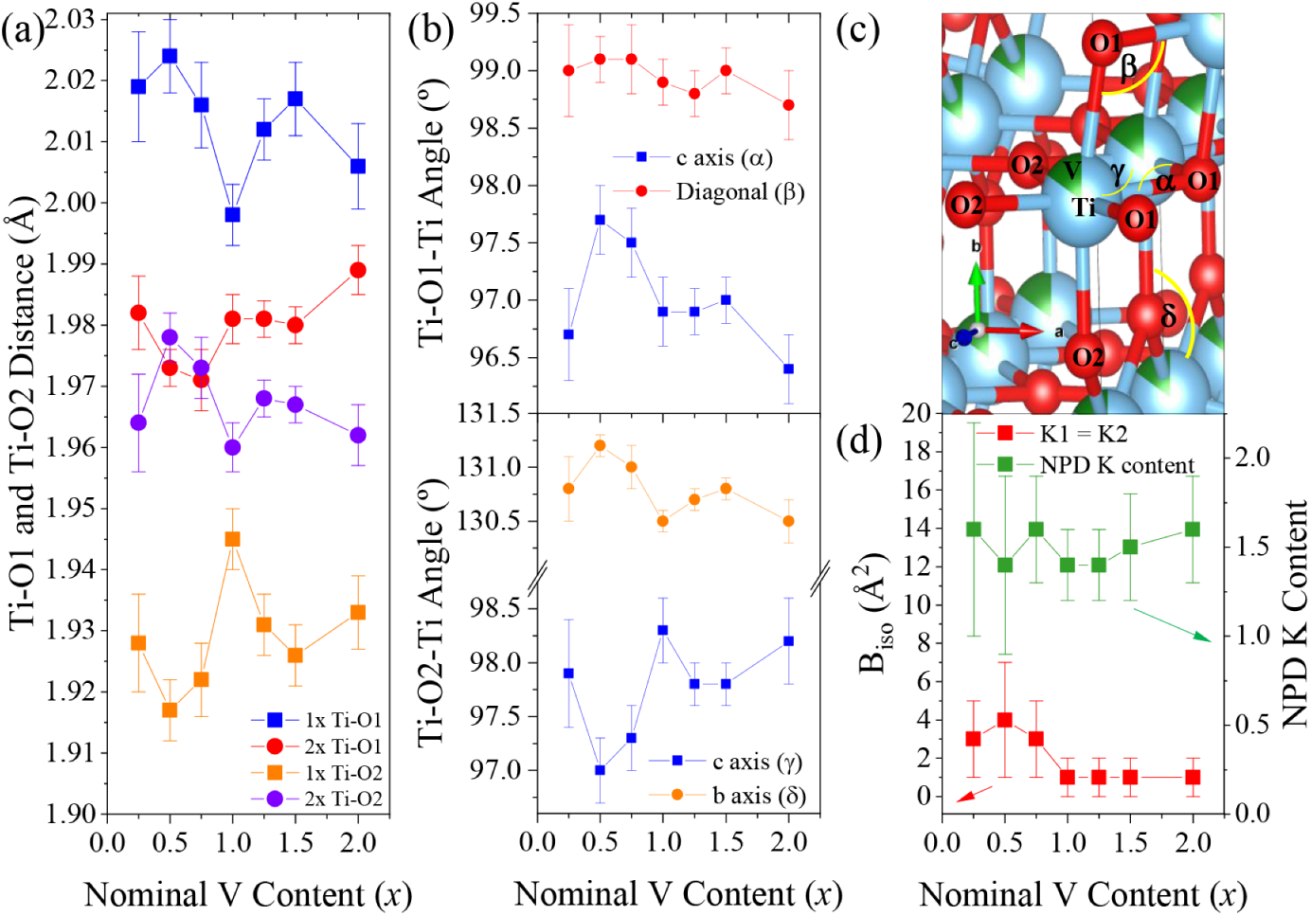
(a) Ti–O1 and Ti–O2 distances *vs* nominal V content for K_*y*_V_*x*_Ti_8__–*x*_O_16_ (0.25 ≤ *x* ≤ 2) samples. (b) Ti–O1–Ti and Ti–O2–Ti angles *vs* nominal V content. (c) Octahedra of (V/Ti)­O_6_ for K_2_V_2_Ti_6_O_16_ nominal composition, where blue spheres are Ti, green spheres are V, and red spheres are O. (d) B_iso_ of K vs nominal V content and NPD K content.

Other important information extracted from neutron data refinement includes the potassium content (which was added in excess for samples with *x* ≤ 1.25), as well as its isotropic atomic displacement parameter (Figure 4d). Regarding K content, these samples exhibit similar amounts of K within their structure, as determined by neutron refinement and in agreement with the reagent mixture. This consistent K content has allowed us to predict a similar average transition metal oxidation state, and to assume that upon increasing the V content, Ti^3+^ is replaced by V^3+^ (as discussed above in relation to average ionic radii and cation polarizing character). Regarding the K (K1 = K2) B_iso_ values, a pronounced drop is observed between nominal compositions with *x* < 1 and those with *x* ≥ 1, which suggests that the reduced K “vibrations” at its tunnel sites decrease as smaller V cations occupy the M_8_O_16_ framework, and the tunnels become narrower. The chemical compositions of the crystalline phase as refined by neutron powder diffraction (Table S1) agree well with that determined by ICP-OES for the *x* = 1.5 composition (Table S2).

### Transmission Electron Microscopies

For each sample, the reciprocal space of the hollandite-type crystals was explored by tilting the selected crystals along different zone axes. These experiments were conducted to seek for additional weak features in the diffraction patterns accompanying the strong Bragg reflections of the underlying average structure.^55^ The presence of diffuse scattering or satellite reflections in the SAED patterns can indicate variations with respect to the ideal average hollandite periodic structure, i.e., additional ordering corresponding to short-range order or to a modulated structure (long-range order, LRO). Moreover, HAADF and ABF-STEM images were acquired to determine the compositional origin associated with the extra weak features that these materials exhibit in their SAED patterns.

Figure 5a shows a high-resolution transmission electron microscopy image taken along the [001] zone axis of the sample with nominal composition K_2_V_2_Ti_6_O_16_. In this image, the tunnels formed by the Ti/VO_6_ double octahedra framework (dark areas) along the *c* axis are clearly visible, within which, the K^+^ ions (dark spots surrounded by bright areas) are accommodated. This image reveals an ordered crystal structure onto which the structural model can be superimposed (inset). The corresponding SAED pattern of this sample, shown in Figure 5b, and the CBED pattern of K_1.75_V_1.75_Ti_6.25_O_16_, shown in Figure 5c, are compatible with a hollandite structure with tetragonal symmetry (*S.G. I*4*/m*, #87) and corroborate the high crystallinity of the samples, with no defects or microstructural effects observed along this zone axis. However, microstructural effects are evident along other crystal directions for several compositions. In particular, for the *x* = 1.5 nominal composition, the electron diffraction pattern along the [010] zone axis evidences satellite reflections at ∼( $00\frac{2}{7}l$ ) with diffuse streak lines parallel to the c* as shown in Figure 5d. Further exploring the reciprocal space shows that the diffuse scattering also extends along the <110>* directions (Figure 5e). This indicates a possible short-range ordering of Ti/V or K/vacancies along the *c* axis within an incommensurately modulated superstructure. The streaking along the <100>* and <110>* directions suggests lateral disorder in the modulation along c*, meaning that there is no perfect correlation in the order along the *c* axis of one unit cell with another. However, upon electrochemical potassium extraction (Figure S1) and chemical potassium insertion (Figure 5f), this intensity disappears, indicating that this extra diffuse intensity is likely related to K-vacancy ordering rather than unusual Ti/V ordering.

**Figure 5:**
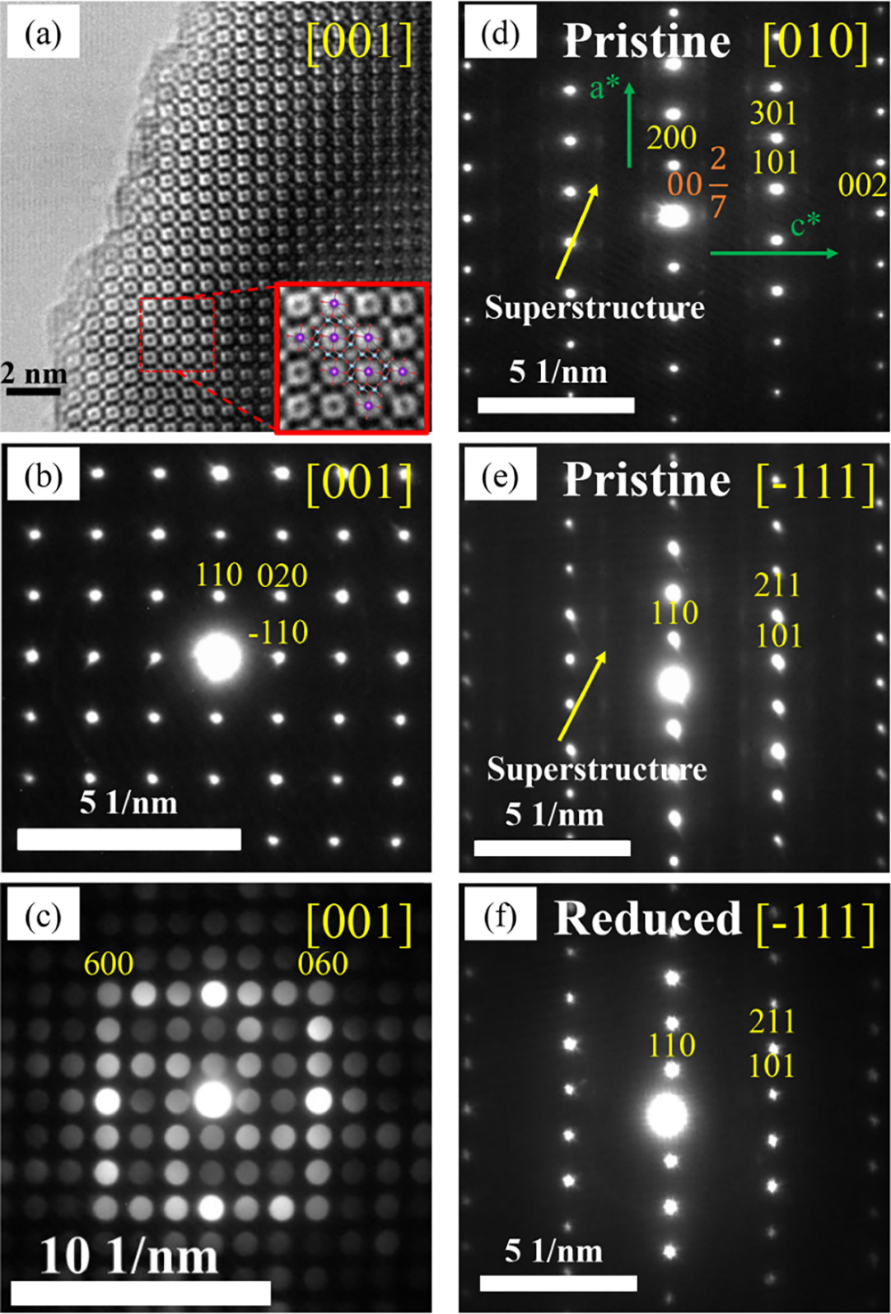
(a) HRTEM micrograph of K_2_V_2_Ti_6_O_16_ taken along the [001] zone axis. A magnified view is provided with the corresponding structural model projection superimposed. Red spheres represent the oxygen atoms, the purple spheres represent potassium, and the blue spheres correspond to V/Ti atoms at the center of the octahedra [V/TiO_6_]. (b) SAED pattern of K_2_V_2_Ti_6_O_16_ along the [001] zone axis. (c) CBED pattern of K_1.75_V_1.75_Ti_6.25_O_16_ along the [001] zone axis. (d) SAED pattern of pristine sample K_1.5_V_1.5_Ti_6.5_O_16_ taken along the [010] zone axis. (e) SAED pattern of pristine sample K_1.5_V_1.5_Ti_6.5_O_16_ taken along the [−111] zone axis. (f) SAED pattern of chemical reduced sample K_1.5_V_1.5_Ti_6.5_O_16_ taken along the [−111] zone axis. SAED patterns (d) and (e) also show a weak, diffuse intensity distribution in addition to the strong Bragg reflections of the average hollandite structure.

These extra weak satellite reflections or diffuse intensity distributions are not exclusive to the *x* = 1.5 material and are also observed in samples with different compositions. SAED patterns of samples with nominal compositions K_2_V_2_Ti_6_O_16_, K_1.5_V_0.75_Ti_7.25_O_16_, and K_1.5_V_0.25_Ti_7.75_O_16_ are shown in Figure 6.

**Figure 6:**
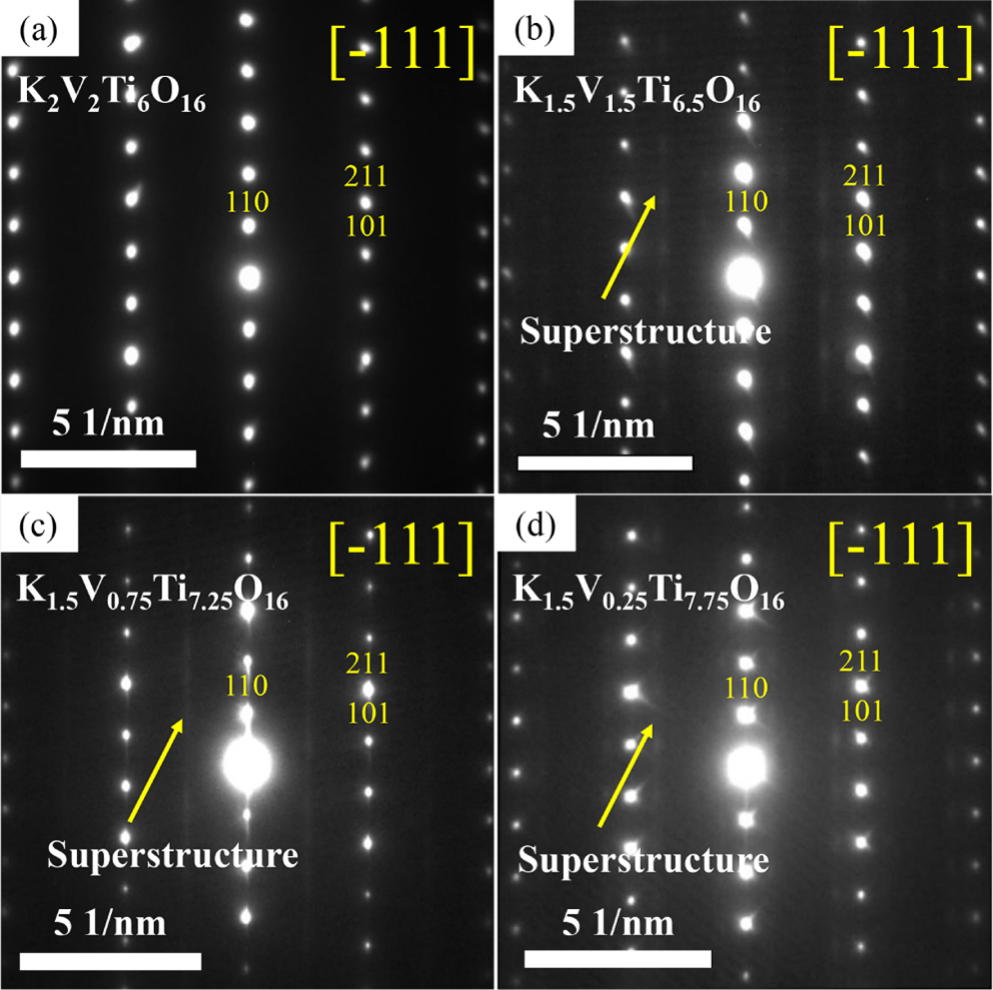
SAED patterns of nominal compositions: (a) K_2_V_2_Ti_6_O_16_, (b) K_1.5_V_1.5_Ti_6.5_O_16_, (c) K_1.5_V_0.75_Ti_7.25_O_16_, and (d) K_1.5_V_0.25_Ti_7.75_O_16_ along the [−111] zone axis. SAED patterns in (b,c,d) also show additional weak, diffuse intensity distribution.

It is noteworthy that among the explored compositions, the one with the nominal composition K_2_V_2_Ti_6_O_16_ (Figure 6a) is the only sample that does not exhibit a superstructure along the [−111] zone axis. The rest of the samples, however, display extra weak satellite reflections and/or diffuse intensity in the SAED patterns taken along the [−111] zone axis, although the appearance of these weak extra features varies. While the samples with nominal compositions K_1.5_V_1.5_Ti_6.5_O_16_ (Figure 6b) and K_1.5_V_0.25_Ti_7.75_O_16_ (Figure 6d) exhibit the same satellite reflections at ( $00\frac{2}{7}l$ ) with diffuse streak lines along the <110>* direction, as mentioned before, the sample with K_1.5_V_0.75_Ti_7.25_O_16_ (Figure 6c) displays a more complex pattern. In this case, some crystals show the same satellite reflections as observed for *x* = 1.5, while others exhibit diffuse streak lines that overlap with reflections along the <110>* direction (e.g., 110, 220, 330) and also appear parallel to them, centered at 1/2*h* 0 1/2*k* reflections with additional streaking. The difference between these two superstructures is likely due to the varying K contents present in different crystals of the sample.

Additionally, particle size analysis was performed by using both TEM and SEM micrographs. TEM measurements on the *x* = 1.5 sample, shown in Figure S2, reveal a particle size of approximately 730 nm for the longest side, which is consistent with SEM image analysis. Figure S3 presents SEM micrographs of the different samples, all showing an agglomerated morphology. The particle size distribution shown in Figure S4 indicates a mean particle size of 610 ± 60 nm for most samples, except for those with nominal compositions of *x* = 2 and *x* = 1.25, which present particle sizes of 2000 ± 1000 and 300 ± 100 nm, respectively. For the *x* = 1.5 sample, SEM analysis shows a particle size of 600 ± 200 nm, aligning well with the TEM results.

### High-Angle Annular Dark-Field Scanning Transmission Electron Microscopy

To rule out any possible Ti/V ordering as the origin of the superstructure observed in the TEM, HAADF-STEM and ABF-STEM micrographs were obtained for crystals showing the most common superstructure reflections in samples with nominal compositions of K_1.5_V_1.5_Ti_6.5_O_16_ and K_1.5_V_0.75_Ti_7.25_O_16_, as shown in Figure 7.

**Figure 7:**
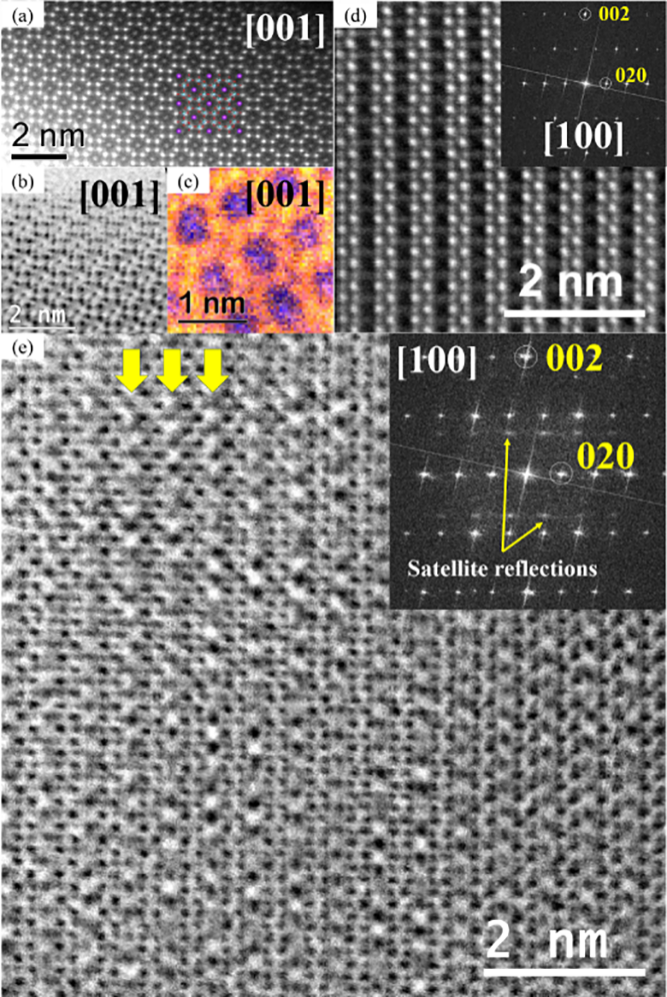
(a) High-resolution HAADF-STEM image of K_1.5_V_1.5_Ti_6.5_O_16_ taken along the [001] zone axis, with a structural overlay model (K, purple; V/Ti, blue; and O, red). (b) High-resolution radial WF-ABF STEM image of K_1.5_V_1.5_Ti_6.5_O_16_ taken along the [001] zone axis. (c) Elemental mapping of Ti L_2,3_ (red), V L_2,3_ (yellow), and K L_2,3_ (blue) of K_1.5_V_1.5_Ti_6.5_O_16_ taken along [001] zone axis. (d) High-resolution radial WF-HAADF image of K_1.5_V_1.5_Ti_6.5_O_16_ along the [100] zone axis, with its corresponding FFT. (e) Processed high-resolution ABF-STEM image of K_1.5_V_0.75_Ti_7.25_O_16_, after enhancing three times the contribution of the satellite reflections, -it shows intensity modulation in the K atomic columns (yellow arrows)-, with its corresponding FFT for the unfiltered image.

Figure 7a presents the [001] zone axis high-resolution HAADF-STEM image of the sample with nominal composition K_1.5_V_1.5_Ti_6.5_O_16_, overlaid with the hollandite-type structure model. In HAADF mode, the heavier atoms appear bright; thus, the tunnels formed by the Ti/VO_6_ double octahedral framework along the *c* axis are clearly visible, with K^+^ ions (also bright) accommodated within the dark regions. The reduced brightness for K *vs* Ti/V, with such similar Z, already suggests not full occupancy of K positions. Alongside this, the [001] zone axis high-resolution radial WF-ABF image (Figure 7b), with nearly inverted contrast as compared to HAADF (dark regions for heavier atoms), and the elemental mapping of Ti, V, and K (Figure 7c) confirm that the tunnels in the hollandite-type framework are occupied by K^+^ ions, with a homogeneous distribution throughout the sample. When looking through the zone axis patterns where the superlattice reflections are observed, major differences are observed between imaging in the HAADF and ABF modes. This could be due to the fact that the intensity of superlattice reflection of compositional origin decreases as the diffraction angle increases,^56,57^ which further supports a compositional modulation (e.g., K/vacancy ordering) as the origin of satellite reflections. Figure 7d depicts the [100] zone axis high-resolution radial WF-HAADF image of a crystal of sample with K_1.5_V_1.5_Ti_6.5_O_16_ nominal composition, where no superstructure is observed; indeed, no superlattice reflections are observed in the FFT of HAADF images. In contrast, satellite reflections are clearly seen in the FFT of ABF images of the same region (Figure S5), and the modulation is thus more clearly seen in their corresponding ABF images. Figure 7e shows a processed high-resolution ABF-STEM image of K_1.5_V_0.75_Ti_7.25_O_16_ enhanced by multiplying the contributions from satellite reflections by three times, thus highlighting intensity modulation within the K columns (yellow arrows, bright areas). Ti/V ordering can thus be definitively excluded, and evidence suggests some form of ordering within the K columns, which lacks long-range order in 3D. This arrangement likely corresponds to two K-occupied sites and one vacancy, or one K-occupied site and two vacancies. Considering a potassium filling exceeding half of the maximum capacity as determined by NPD, XRF, and EDS-SEM (Table S1), two occupied sites and one vacancy align more closely with the observed structure. Notably, the superstructure, although very frequently observed, is not consistently present in all studied crystals. Indeed, including a modulation vector *q* = $\left(00\frac{2}{7}\right)$ in the Rietveld refinement of the NPD pattern did not result in a satisfactory fit. Alternatively, adding a second phase with the modulation vector did not lead to a significant phase fraction. These findings suggest that the superstructure has a local, non long-range origin.

### Magnetic Properties

The ZFC-FC plots of (*χ*_T_–*χ*_D_) vs *T* and (*χ*_T_–*χ*_D_)^−1^ vs *T* for nominal composition K_2_V_2_Ti_6_O_16_ are shown in Figure 8a,b, respectively as a representative example. The ZFC-FC plots for other compositions (0.25 ≤ *x* ≤ 1.5) are provided in Figure S6. For the *x* = 1.5 nominal composition, a diamagnetic susceptibility of −2.64 × 10^–4^ emu·mol^–1^·Oe^–1^ was calculated. This value aligns well with the diamagnetic susceptibility reported in a previous study.^53^ The slight deviation in this value of diamagnetic susceptibility is likely due to the slight compositional differences between their single crystals and our polycrystalline materials. Moreover, for all these compositions, except for K_1.5_V_0.25_Ti_7.75_O_16_, no splitting of the ZFC-FC curve is observed, suggesting that these samples exhibit paramagnetic behavior in the temperature range between 2 and 300 K.

**Figure 8:**
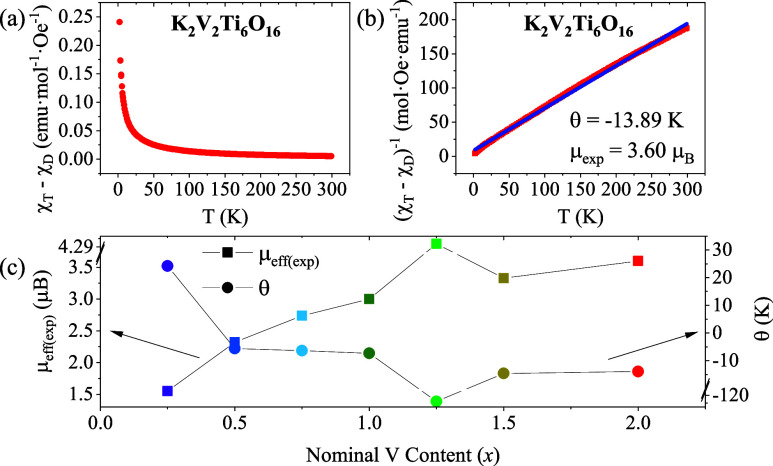
(a) Paramagnetic susceptibility (*χ*_T_–*χ*_D_) vs temperature (*T*) and (b) Inverse of the paramagnetic susceptibility (*χ*_T_–*χ*_D_)^−1^ vs temperature (*T*) for K_2_V_2_Ti_6_O_16_ sample. The linear fitting is also shown in figure b (blue). (c) Experimental magnetic moment (μ_exp_, square symbols) and Weiss constant (θ, circles) vs nominal V content (*x*) for the prepared compositions.

Samples with nominal compositions *x* = 2, 1.5, 1, 0.75, and 0.5 behave as paramagnetic across the entire linear temperature range, except for low temperatures (2–50 K) where there are magnetic interactions. The magnetic moment (μ_exp_) and Weiss constant (θ) for all compositions were determined from the plot and fitting of (*χ*_T_–*χ*_D_)^−1^ vs *T* (Curie–Weiss law, eq 1), as illustrated in Figure 8c and summarized in Table 2.

**Table 2:** Magnetic Moment (μ_exp_) and Weiss Constant (θ) Calculated for the Synthesized Samples from the Fitting of (*χ*_T_–*χ*_D_)^−1^ vs *T* (Curie-Weiss law)

K*_y_* V*_x_* Ti_8**–** *x*_O_16_					
*x* Nominal	NPD Composition	Curie Constant, *C* (emu·K·mol^–1^·Oe^–*1*^ *)*	Magnetic Moment, μ_exp_ (μ_B_)	Weiss Constant, θ (K)	Calculated Ti^3+^(%)
2	K_1.6(3)_V_1.3(3)_Ti_6.7(3)_O_16_	1.62	3.60	–13.8	12.7
1.5	K_1.5(3)_V_1.3(3)_Ti_6.7(3)_O_16_	1.39	3.33	–14.6	3.4
1.25	K_1.4(2)_V_0.8(3)_Ti_7.2(3)_O_16_	2.37	4.35	–128	58
1	K_1.4(2)_V_0.8(2)_Ti_7.2(2)_O_16_	1.13	3.00	–7.3	12.0
0.75	K_1.6(3)_V_0.8(3)_Ti_7.2(3)_O_16_	0.94	2.74	–6.4	5.1
0.5	K_1.4(5)_V_0.2(3)_Ti_7.8(3)_O_16_	0.67	2.32	–5.6	16.2
0.25	K_1.6(6)_V_0.3(3)_Ti_7.7(3)_O_16_	0.30	1.55	24.2	0

Most compositions show antiferromagnetic interactions, as indicated by the negative values of θ. For samples with nominal compositions *x* = 1.25 and 0.25, a linear fit to the (*χ*_T_–*χ*_D_)^−1^ vs *T* plot is possible only at high temperatures: 200–295 K for K_1.5_V_1.25_Ti_6.75_O_16_ and 200–270 K for K_1.5_V_0.25_Ti_7.75_O_16_, thus obtaining μ_exp_ = 4.35 and 1.55 μ_B_ and Weiss constants (θ) = −128 and 24.2 K, respectively. These results suggest that K_1.5_V_1.25_Ti_6.75_O_16_ also exhibit antiferromagnetic interactions at low temperatures, whereas in K_1.5_V_0.25_Ti_7.75_O_16_ ferromagnetic interactions are taking place. For the *x* = 1.5 nominal composition, a μ_exp_ = 3.33 μ_B_ is obtained. This value is also in good terms with μ_exp_ determined in Moetakef et al.’s previous work.^53^ The slightly larger value is due to actual different compositions of both materials K_1.5_V_1.5_Ti_6.5_O_16_ vs K_1.5_V_1.3_Ti_6.7_O_16_, our material having some Ti^3+^ content, whereas all Ti is 4+ in Moetakeḟ et al.’s work. An inverse correlation between the μ_exp_ and θ is seen in Figure 8c: as μ_exp_ increases with the nominal V content, θ tends to decrease, and vice versa. The increase in μ_exp_ with increasing *x* is expected due to the higher magnetic moment of V^3+^ (d^2^) and V^4+^ (d^1^) compared to Ti^3+^(d^1^) and Ti^4+^ (d^0^—diamagnetic). The experimental magnetic moment (μ_exp_) of the materials provides a basis for estimating the oxidation states of V and Ti (Table S3). Assuming all V is in +3 oxidation state (V^3+^), the amount of Ti^3+^ can be calculated (eq S1), and thus an average oxidation state of Ti, as shown in Table S3. The calculated percentage of Ti^3+^ in the samples varies from 3.4% to 58%, except for *x* = 0.25, which is practically zero (0.01%). This value suggests that this composition contains no Ti^3+^ and all the Ti exhibits a + 4 oxidation state (Ti^4+^).

### Electrochemical Performance

The electrochemical performance of these as-synthesized hollandites as electrode materials in KIBs was studied through galvanostatic charge–discharge measurements. Using K_2_V_2_Ti_6_O_16_ nominal composition as a starting point, parameters such as the electrolyte composition and potential window were optimized for the rest of the compositions.

The electrochemical study of *x* = 2 was initially conducted in a potential window between 0.9 and 2 V and using 3.9 M KFSI (DME) as electrolyte (Figure S7). Due to the low capacity reached and the insertion-extraction of so few K^+^, the material was studied in a wider potential window from 0.9 to 3.8 V, as shown in Figure 9a (Figure S8). As it can be seen, in the first discharge process, the material exhibits a large plateau starting at ∼1.2 V, reaching a capacity close to 130 mA·h·g^–1^. This capacity exceeds the theoretical value for the exchange of 2 K^+^, which is 74 mA·h·g^–1^, although most of the reaction is irreversible. Therefore, this plateau is not only attributed to ion insertion processes but may also result from further conversion reactions, which is discarded as it would involve large structural changes that are not observed (Figure S9), or from electrolyte decomposition, including the formation of the solid electrolyte interphase (SEI) layer during the first cycle. The SEI layer, while passively degrading the electrolyte, should facilitate ion conduction and thereby enable reversible cycling of the material. However, in the first charge process, only 0.28 K^+^ per formula unit are extracted. Nonetheless, in cycle 2 and after the intercalation of more K^+^, reversible insertion and extraction of 0.81 K^+^/f.u. are achieved. Cycling again at a rate of C/10 (cycle 25) revealed a decrease in ion exchange, with 0.61 K^+^ ions being inserted and extracted, indicating a gradual reduction in ion exchange with continued cycling.

**Figure 9:**
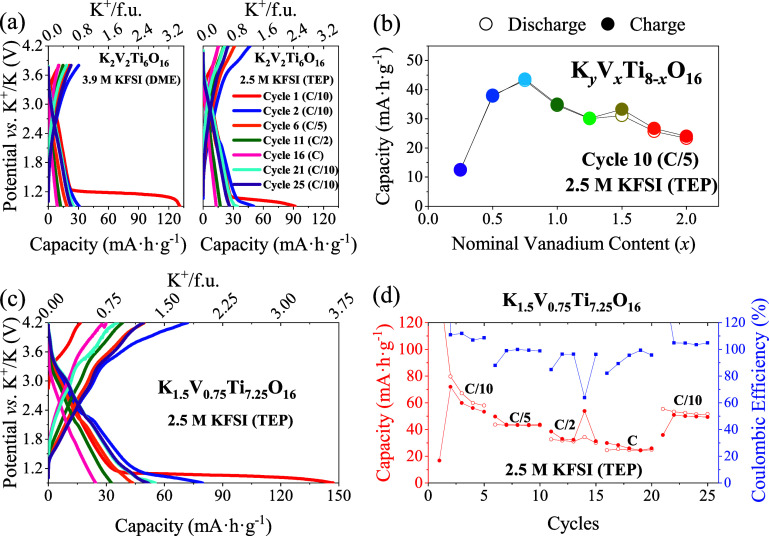
(a) Galvanostatic charge–discharge comparison between two nominal composition K_2_V_2_Ti_6_O_16_ cells using two different electrolytes. (b) Capacity reached during galvanostatic charge–discharge of cycle 10 of the different nominal compositions when cycled at C/5. (c) Galvanostatic charge–discharge of the nominal composition K_1.5_V_0.75_Ti_7.25_O_16_. (d) Capacity vs Coulombic efficiency during galvanostatic cycling of the sample with K_1.5_V_0.75_Ti_7.25_O_16_ nominal composition.

After establishing the optimal potential window for the nominal composition K_2_V_2_Ti_6_O_16_ with 3.9 M KFSI (DME), its performance was compared to that in 2.5 M KFSI in triethylphosphate (TEP). The latter electrolyte allowed the potential window to be extended to 4.2 V vs K^+^/K without inducing oxidative decomposition of the electrolyte, as shown in Figure 9a (right).

In the first cycle, a large plateau is formed again at approximately 1.2 V, which reaches higher capacities (150 mA·h·g^–1^) than the theoretical capacity of the sample, and the second cycle reaches 50 mA·h·g^–1^ with reversible extraction and insertion of 1.2 K^+^/f.u. An improvement in capacity recovery is also observed in cycle 21 when using the 2.5 M KFSI (TEP) electrolyte. Thus, it can be stated that cells using 2.5 M KFSI (TEP) as electrolyte reach higher reversible capacity, as it is stable up to 4.2 V (Figure S10), than cells cycling with 3.9 M KFSI (DME), where the electrolyte is oxidized above 3.8 V (Figure S10). Therefore, 2.5 M KFSI (TEP) was the chosen electrolyte to study other nominal compositions. Figure 9b shows the values of capacity obtained during galvanostatic charge–discharge at cycle 10 (when cycled at C/5) for materials with different nominal compositions (0.25 ≤ *x* ≤ 2). Capacity reaches higher values as the nominal vanadium content increases up to a maximum of *x* = 0.75, where K B_iso_ is still large and different superstructure reflections appear. Reversible capacity then decreases for larger vanadium contents, where the tunnel size decreases and K B_iso_ drops. The K_1.5_V_0.25_Ti_7.75_O_16_ nominal composition shows the lowest reversible capacity. Figure 9c exhibits the galvanostatic charge–discharge curves for the K_1.5_V_0.75_Ti_7.25_O_16_ nominal composition at different C rates. As usual, at 1.2 V a large plateau is formed, as it happened for all nominal compositions, reaching a total capacity of 150 mA·h·g^–1^. Figure 9d shows the capacity values reached for the K_1.5_V_0.75_Ti_7.25_O_16_ nominal composition during cycling and the Coulombic efficiency values obtained for each cycle. A decrease in capacity in the first five cycles can also be seen. The discharge capacity of cycle 2 is 80 mA·h·g^–1^ (insertion of 2 K^+^/f.u.), while the charge capacity reaches 72 mA·h·g^–1^ (extraction of 1.80 K^+^/f.u.). However, for cycle 5 the discharge capacity is 58 mA·h·g^–1^ (insertion of 1.45 K^+^/f.u.) and the charge capacity is 53 mA·h·g^–1^ (extraction of 1.33 K^+^/f.u.). The Coulombic efficiency values calculated for C/10 rates are 107 ± 3%, while for C/2 and C rates are 87 ± 14% and 92 ± 7%, respectively. For cycles at C/5 rate, the Coulombic efficiency values are 97 ± 5%. After cycling at the C rate and cycling again at C/10, the capacity increases to 53 mA·h·g^–1^ and it remains stable until the end of cycling.

To compare these electrochemical results for the use of K*_y_* V*_x_* Ti_8–*x*_O_16_ hollandite-type materials with 0.25 ≤ *x* ≤ 2 nominal composition in KIBs with that reported for K_0.17_TiO_2_ hollandites,^49^ we attempted to study our material with *x* = 0.75 nominal composition using 0.5 M KFSI (EC:DEC) under the same measurement conditions as employed by Jo et al.^49^ However, the capacities obtained using 0.5 M KFSI (EC:DEC) were lower than the ones obtained with our electrolyte, 2.5 M KFSI (TEP) (Figure S11). Furthermore, the capacity retention of the material after 12 cycles, varying the C rate, using 2.5 M KFSI (TEP) is 63%, while using 0.5 M KFSI (EC:DEC) is 60% (Figure S11). Thus, using 2.5 M KFSI (TEP) as electrolyte, we achieved initial reversible capacity similar to those of undoped K_0.17_TiO_2_ (80 mA·h·g^–1^), but a lower reversible capacity of 50 mA·h·g^–1^ after 20 cycles studied at different C rates, compared to their 60 mA·h·g^–1^ after 1000 cycles at 5 C. This decrease of capacity with increased V substitution, particularly at high rates, could be related to the decreased K B_iso_, which implies more tightly bound K-ions, along with a narrowing of the tunnels as the V content increases, as well as the resulting formation of superstructures (due to K/vacancy ordering), which has not been explored for V-undoped compositions.

## Conclusions

New hollandite phases of nominal composition K*_y_* V*_x_* Ti_8–*x*_O_16_ (0.25 ≤ *x* ≤ 2) have been successfully synthesized by the citrate method. All phases exhibit the tetragonal symmetry of the aristotype hollandite, *I*4*/m,* as confirmed by PXRD and NPD. Rietveld refinement of NPD, combined with chemical composition from XRF and EDS-SEM, suggests a similar K content for all hollandite-type phases with values *y* ≈ 1.4–1.6. TEM analyses confirmed the crystalline structure of the synthesized samples and revealed an additional ordering along the *c* axis attributed to K/vacancy ordering within the tunnels, as confirmed by HAADF-STEM. Diffuse scattering along the <100>* and <110>* further indicates short-range ordering within tunnels across different unit cells. Different satellite reflections and/or diffuse scattering were observed in many crystals of most phases, but their intensity diminished or disappeared entirely upon electrochemical oxidation or chemical reduction. Magnetic characterization demonstrated that the samples exhibit paramagnetic behavior at room temperature with antiferromagnetic interactions emerging at low temperatures, except for the K_1.5_V_0.25_Ti_7.75_O_16_ composition, where ferromagnetic interactions are suggested. Based on the experimentally determined magnetic moments in the paramagnetic regime, potassium and vanadium contents derived from NPD, and under the assumption that all vanadium ions are in the +3 oxidation state (V^3+^), the Ti^3+^ content was estimated to range from 3.4–16.2% for most samples. These K_1.5_V*_x_* Ti_8–*x*_O_16_ phases were able to reversibly insert and extract up to 2 K^+^/f.u. during the second cycle at C/10 within a voltage window of 0.9–4.2 V, using 2.5 M KFSI in TEP as electrolyte. At higher currents, C/5, a maximum of one K-ion was reversibly inserted and extracted for the sample with nominal composition K_1.5_V_0.75_Ti_7.25_O_16_, showing their potential to work as electrodes in potassium-ion batteries.

## Supplementary Material



## Notes

Composition obtained via XRF and EDS-SEM, ICP-OES, and TEM analysis, SEM micrographs and particle size distribution graph, ABF-STEM and HAADF-STEM, with their corresponding FFT collected simultaneously on *x* = 1.5, inverse of paramagnetic susceptibility vs temperature graph, table of calculated Ti^3+^%, and electrochemical performance graphs with PXRD pattern comparison between pristine and reduced sample (PDF)
