# Supplementary material for: Exploring Hollandite-Type K y V x Ti8–x O16 (0.25 ≤ x ≤ 2) as Electrode Materials in Potassium-Ion Batteries (KIBs)
Source: Inorg Chem. 2025 Apr 22;64(17):8578–90. doi: 10.1021/acs.inorgchem.4c05579 (PMC12123605; doi:10.1021/acs.inorgchem.4c05579)
Supplement: Supplementary file 1 [file ic4c05579_si_001.pdf]

Supporting Information

Exploring hollandite-type $K_yV_xTi_{8-x}O_{16}$ (0.25 ≤ x ≤ 2) as electrode materials in potassium ion batteries (KIBs)

Juan Andrés Nieto-Simón[a], Marta María González-Barrios[a], Adrián Gómez-Herrero[b], María Teresa Fernández-Díaz[c], Jesús Prado-Gonjal[a,*], Elizabeth Castillo-Martínez[a,*]

[a]Departamento de Química Inorgánica, Facultad de Ciencias Químicas, Universidad Complutense de Madrid, E-28040 Madrid, Spain

[b]ICTS Centro Nacional de Microscopía Electrónica, Universidad Complutense de Madrid, E-28040 Madrid, Spain

[c]Institut Laue Langevin, Grenoble, F-38042, France

[*]Corresponding authors. Email addresses: ecastill@ucm.es (Elizabeth Castillo-Martínez), jpradogo@ucm.es (Jesús Prado-Gonjal).

X-Ray Fluorescence (XRF) and Energy Dispersive X-Ray Spectroscopy – Scanning Electron Microscopy (EDS-SEM)

Given the similar K content for all compositions extracted from neutron diffraction data, XRF and EDS-SEM measurements were also conducted for these samples (Table S1), which give us a similar overall sample composition.

Table S1. Comparison between nominal, NPD, XRF and EDS-SEM compositions.

$K_yV_xTi_{8-x}O_{16}$			
Nominal Composition	*NPD Composition*	*XRF Composition*	*EDS-SEM Composition*
$K_2V_2Ti_6O_{16}$	$K_{1.6(3)}V_{1.3(3)}Ti_{6.7(3)}O_{16}$	$K_{1.40}V_{1.86}Ti_{6.14}O_{16}$	$K_{1.7(2)}V_{1.5(2)}Ti_{6.5(2)}O_{16}$
$K_{1.75}V_{1.75}Ti_{6.25}O_{16}$		$K_{1.46}V_{1.73}Ti_{6.27}O_{16}$	$K_{1.6(1)}V_{1.5(5)}Ti_{6.5(5)}O_{16}$
$K_{1.5}V_{1.5}Ti_{6.5}O_{16}$	$K_{1.5(3)}V_{1.3(3)}Ti_{6.7(3)}O_{16}$	$K_{1.36}V_{1.42}Ti_{6.58}O_{16}$	$K_{1.6(1)}V_{1.5(1)}Ti_{6.5(1)}O_{16}$
$K_{1.5}V_{1.25}Ti_{6.75}O_{16}$	$K_{1.4(2)}V_{0.8(3)}Ti_{7.2(3)}O_{16}$	$K_{1.08}V_{1.21}Ti_{6.79}O_{16}$	$K_{1.2(2)}V_{1.2(2)}Ti_{6.8(2)}O_{16}$
$K_{1.5}VTi_7O_{16}$	$K_{1.4(2)}V_{0.8(2)}Ti_{7.2(2)}O_{16}$	$K_{1.30}V_{1.29}Ti_{6.71}O_{16}$	$K_{2.0(4)}V_{1.2(2)}Ti_{6.8(2)}O_{16}$
$K_{1.5}V_{0.75}Ti_{7.25}O_{16}$	$K_{1.6(3)}V_{0.8(3)}Ti_{7.2(3)}O_{16}$	$K_{1.31}V_{1.04}Ti_{6.96}O_{16}$	$K_{1.7(3)}V_{1.0(3)}Ti_{7.0(3)}O_{16}$
$K_{1.5}V_{0.5}Ti_{7.5}O_{16}$	$K_{1.4(5)}V_{0.2(3)}Ti_{7.8(3)}O_{16}$	$K_{1.00}V_{0.63}Ti_{7.37}O_{16}$	
$K_{1.5}V_{0.25}Ti_{7.75}O_{16}$	$K_{1.6(6)}V_{0.3(3)}Ti_{7.7(3)}O_{16}$		$K_{1.4(1)}V_{0.4(2)}Ti_{7.6(2)}O_{16}$

It should be noted that XRF is a semi-quantitative analysis of the bulk of the sample and EDS-SEM is an analysis of the composition of the surface of the sample, while NPD can accurately determine the composition of a specific phase contributing to the Bragg reflections. In general, the experimental content of K in the samples determined using XRF is lower than the one obtained via NPD and EDS-SEM and the V content evolves as expected. This suggests the presence of some non-K containing impurity.

Inductively Coupled Plasma – Optical Emission Spectroscopy (ICP-OES)

Table S2. Results of ICP-OES analysis for $K_{1.5}V_{1.5}Ti_{6.5}O_{16}$ sample.

Replicate	Ti (%)	V (%)	K (%)	ICP Composition	Average ICP Composition
x = 1.5 (1)	69.4	14.3	13.7	$K_{1.62}V_{1.30}Ti_{6.70}O_{16}$	$K_{1.56(5)}V_{1.29(1)}Ti_{6.71(1)}O_{16}$
x = 1.5 (2)	60.0	12.2	11.1	$K_{1.52}V_{1.28}Ti_{6.72}O_{16}$	
x = 1.5 (3)	60.4	12.4	11.4	$K_{1.55}V_{1.29}Ti_{6.71}O_{16}$	

Transmission Electron Microscopy

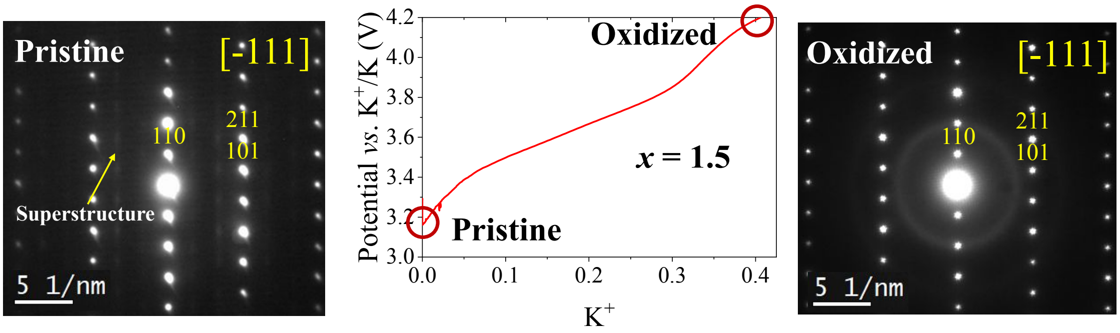

Figure S1. Evolution of superstructure reflections during electrochemical oxidation. [-111] ZAP pattern of x = 1.5 nominal composition before (left) and after (right) the electrochemical oxidation process shown in the center. All superstructure reflections are lost after K removal which confirms its origin to be due to the presence of K ions. Moreover, an amorphous ring is observed in the electrochemically oxidized sample due to surrounding amorphous carbon and electrolyte residues which had been mixed with the hollandite active material for electrochemical cycling.

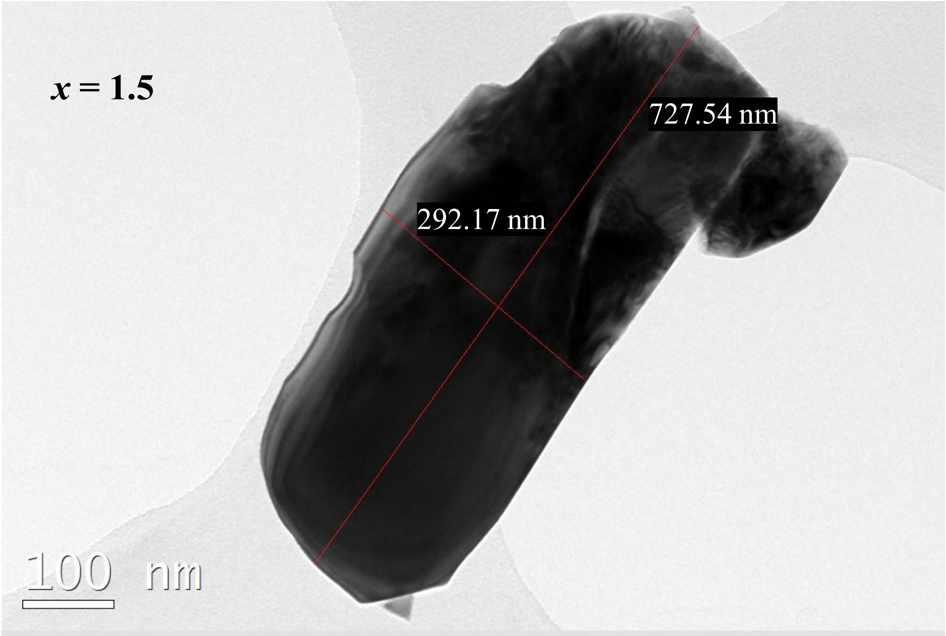

Figure S2. HRTEM micrograph of $K_{1.5}V_{1.5}Ti_{6.5}O_{16}$ sample.

Scanning Electron Microscopy

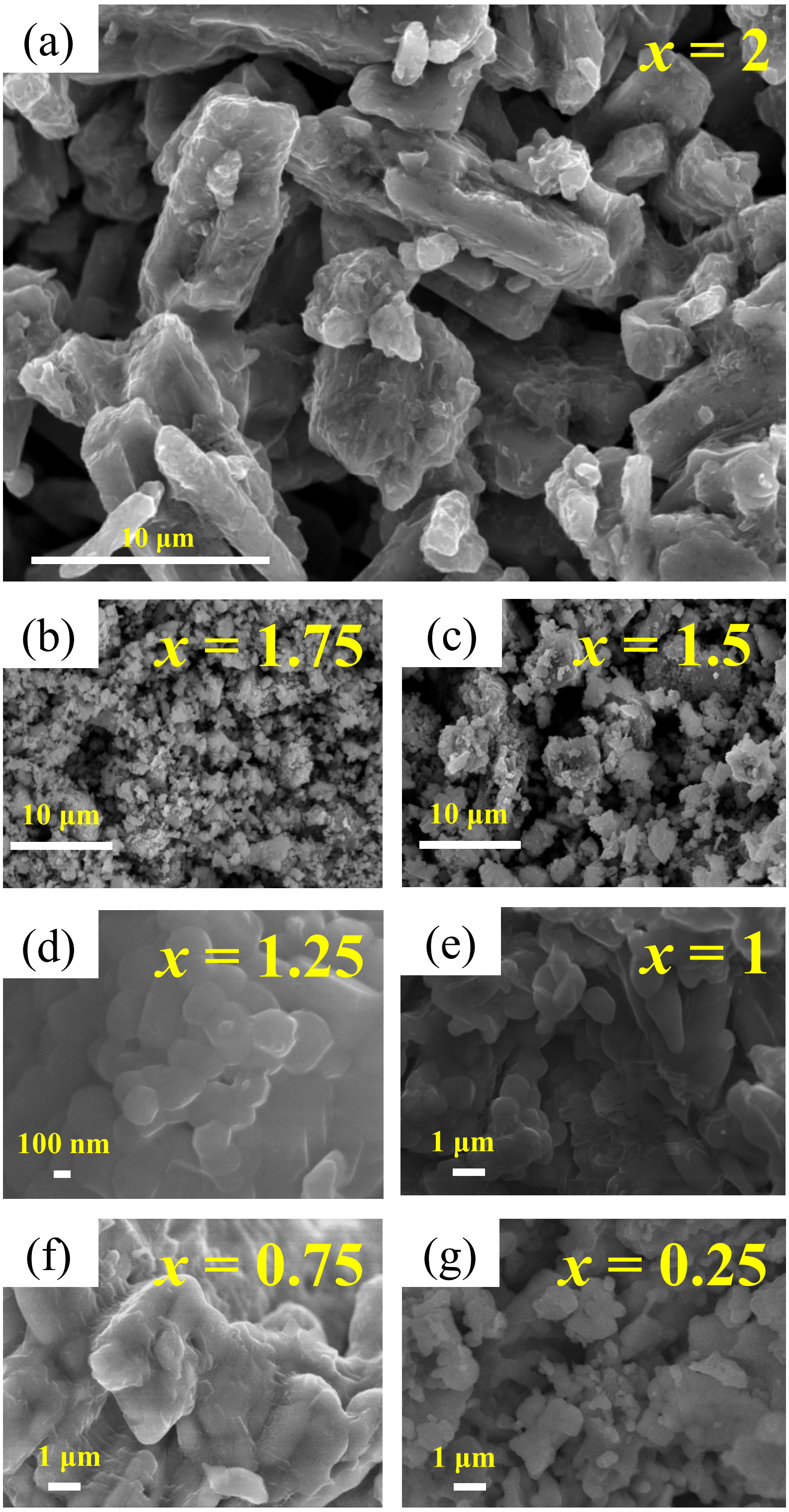

Figure S3. SEM micrographs of $K_yV_xTi_{8-x}O_{16}$ nominal compositions where (a) $x = 2$, (b) $x = 1.75$, (c) $x = 1.5$, (d) $x = 1.25$, (e) $x = 1$, (f) $x = 0.75$ and (g) $x = 0.25$.

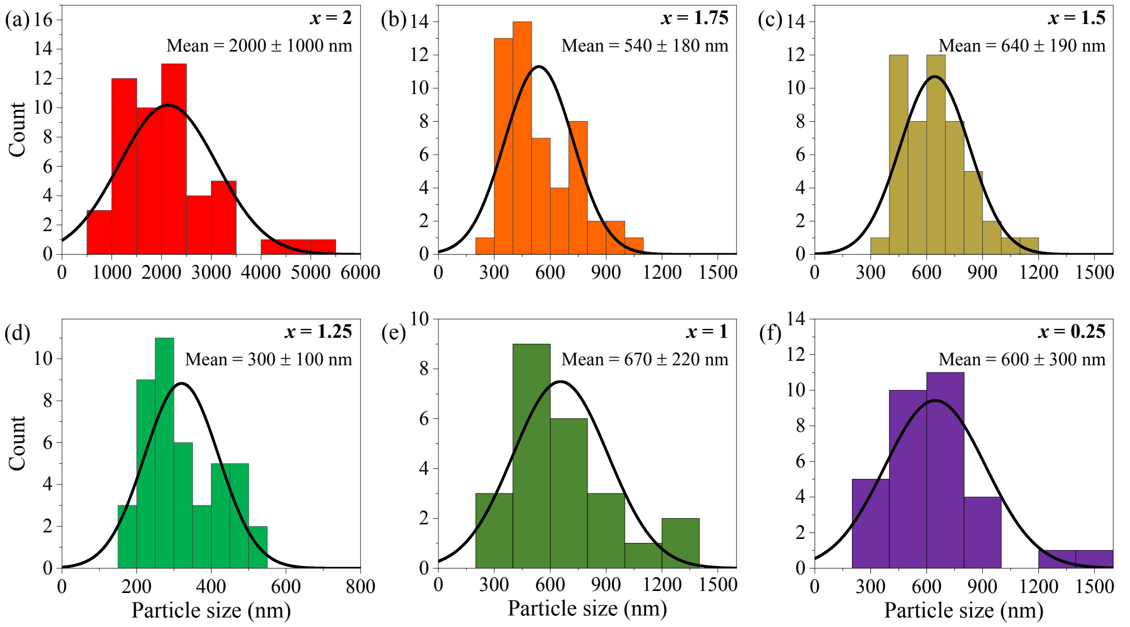

Figure S4. Particle size distribution of $K_yV_xTi_{8-x}O_{16}$ nominal compositions where (a) x = 2, (b) x = 1.75, (c) x = 1.5, (d) x = 1.25, (e) x = 1 and (f) x = 0.25.

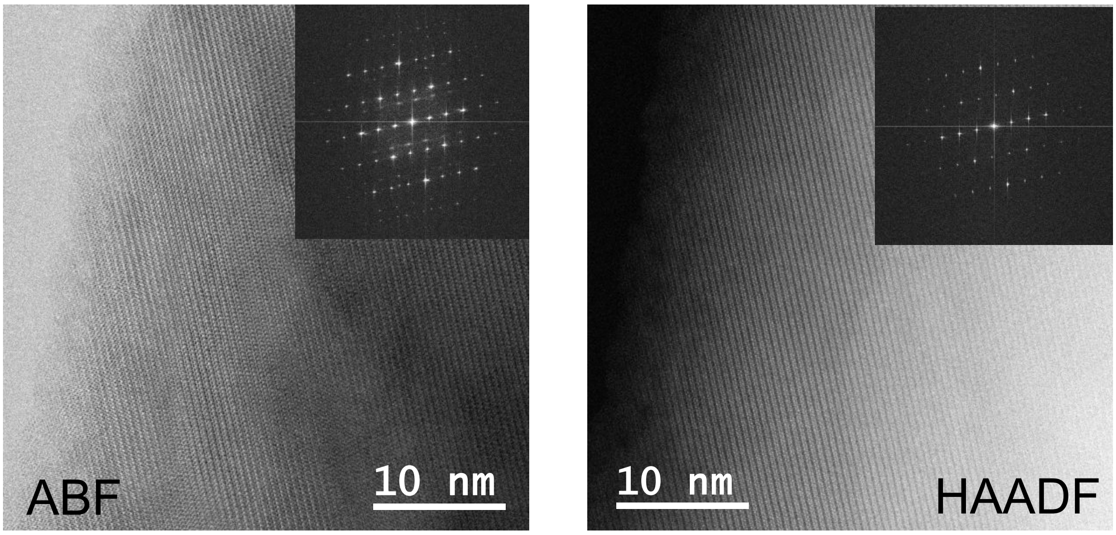

Figure S5. STEM-ABF and STEM-HAADF images (along with their corresponding FFT) collected simultaneously along the [100] ZAP for the hollandite with nominal composition $K_{1.5}V_{1.5}Ti_{6.5}O_{16}$. Satellite reflections are clearly seen in the ABF mode and not in the HAADF mode.

Magnetic Properties

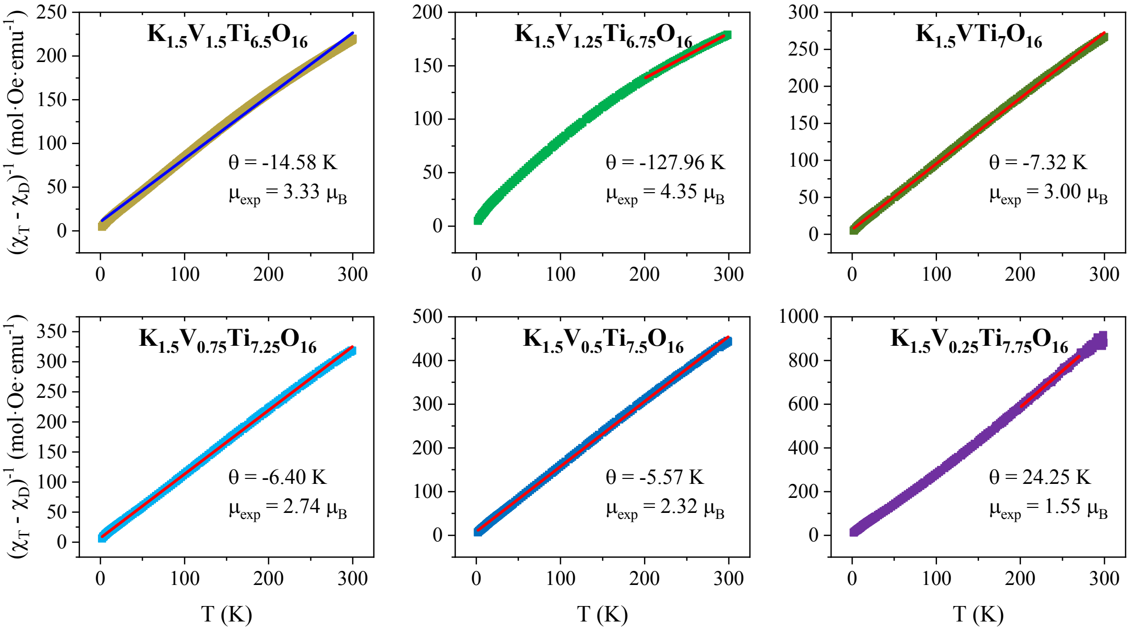

Figure S6. Inverse of the FC-ZFC paramagnetic susceptibility ~~(χ^{-1})~~ *vs*. temperature for $K_yV_xTi_{8-x}O_{16}$ nominal compositions. The regression lines, Weiss constant (θ) and μ_{exp} calculated for every sample are also shown.

Table S3. Different assumptions based on the possible oxidation states of the transition elements V and Ti in each as-synthesized composition. Assuming all V is V^{3+}.

**Composition**			**μ_{exp} (μ_B)**	**μ_{calc} (μ_B)**				**Calculated Ti^{3+}%**
***x* nominal**	**NPD**	**XRF**		*Ti^{3+} 0%*	*Ti^{3+} 5%*	*Ti^{3+} 10%*	*Ti^{3+} 15%*	
2	$K_{1.6(3)}V_{1.3(3)}Ti_{6.7(3)}O_{16}$	$K_{1.40}V_{1.86}Ti_{6.14}O_{16}$	**3.60**	3.22	3.38	3.52	3.66	12.7
1.5	$K_{1.5(3)}V_{1.3(3)}Ti_{6.7(3)}O_{16}$	$K_{1.36}V_{1.42}Ti_{6.58}O_{16}$	**3.33**	3.22	3.38	3.52	3.66	3.4
1.25	$K_{1.4(2)}V_{0.8(3)}Ti_{7.2(3)}O_{16}$	$K_{1.08}V_{1.21}Ti_{6.79}O_{16}$	**4.35**	2.53	2.73	2.93	3.10	58.0
1	$K_{1.4(2)}V_{0.8(2)}Ti_{7.2(2)}O_{16}$	$K_{1.30}V_{1.29}Ti_{6.71}O_{16}$	**3.00**	2.53	2.73	2.93	3.10	12.0
0.75	$K_{1.6(3)}V_{0.8(3)}Ti_{7.2(3)}O_{16}$	$K_{1.31}V_{1.04}Ti_{6.96}O_{16}$	**2.74**	2.53	2.73	2.93	3.10	5.1
0.5	$K_{1.4(5)}V_{0.2(3)}Ti_{7.8(3)}O_{16}$	$K_{1.00}V_{0.63}Ti_{7.37}O_{16}$	**2.32**	1.26	1.66	1.98	2.26	16.2
0.25	$K_{1.6(6)}V_{0.3(3)}Ti_{7.7(3)}O_{16}$		**1.55**	1.55	1.88	2.17	2.42	0.01

The amount of Ti^{3+} was calculated according to equation S1:

$$\frac{a}{100}\cdot x\cdot\mu^2(V^{3+}) + \frac{b}{100}\cdot(8-x)\cdot\mu^2(Ti^{3+}) = (\mu_{exp})^2 \qquad \text{(Eq. S1)}$$

where a and b are the percentages of V^{3+} and Ti^{3+} in the sample, respectively. Assuming all V is V^{3+}, a will be 100 for all cases. Parameter x is the amount of V in the sample, given by the subscripts of the NPD compositions, and μ is the magnetic moment.

Magnetic moments (μ) for V^{3+} and Ti^{3+} are calculated as spin-only values as follows:

$$\mu_{s.o.} = \sqrt{n(n+2)} \qquad \text{(Eq. S2)}$$

where n is the number of unpaired electrons of V^{3+}, which is 2, and Ti^{3+}, which is 1.

Experimental magnetic moment (μ_{exp}) is calculated according to equation S3:

$$\mu_{exp} = \sqrt{8 \cdot C} \qquad \text{(Eq. S3)}$$

where C is the Curie constant ($emu \cdot K \cdot mol^{-1} \cdot Oe^{-1}$) calculated from Curie Weiss fittings (Figure S6) of experimental data according to equation 2 (main text).

Electrochemical Properties

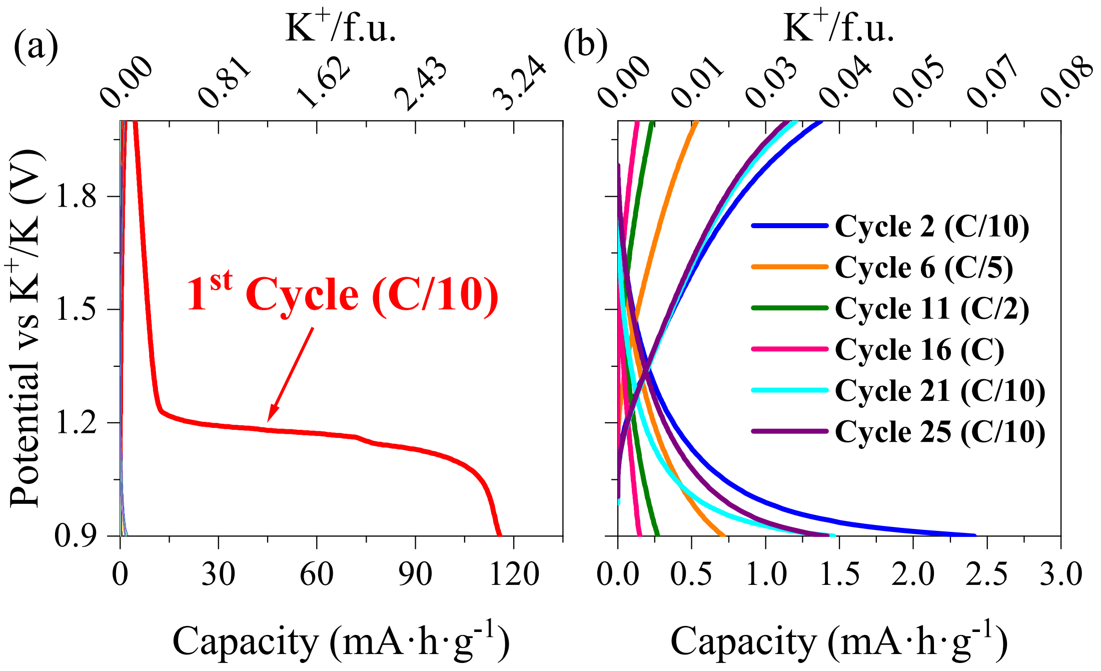

Figure S7. (a) Galvanostatic charge-discharge curves for sample with $K_2V_2Ti_6O_{16}$ nominal composition studied in a potential window between 0.9 – 2 V using 3.9 M KFSI (DME) as electrolyte. (b) Enlargement of image (a), without representing cycle 1.

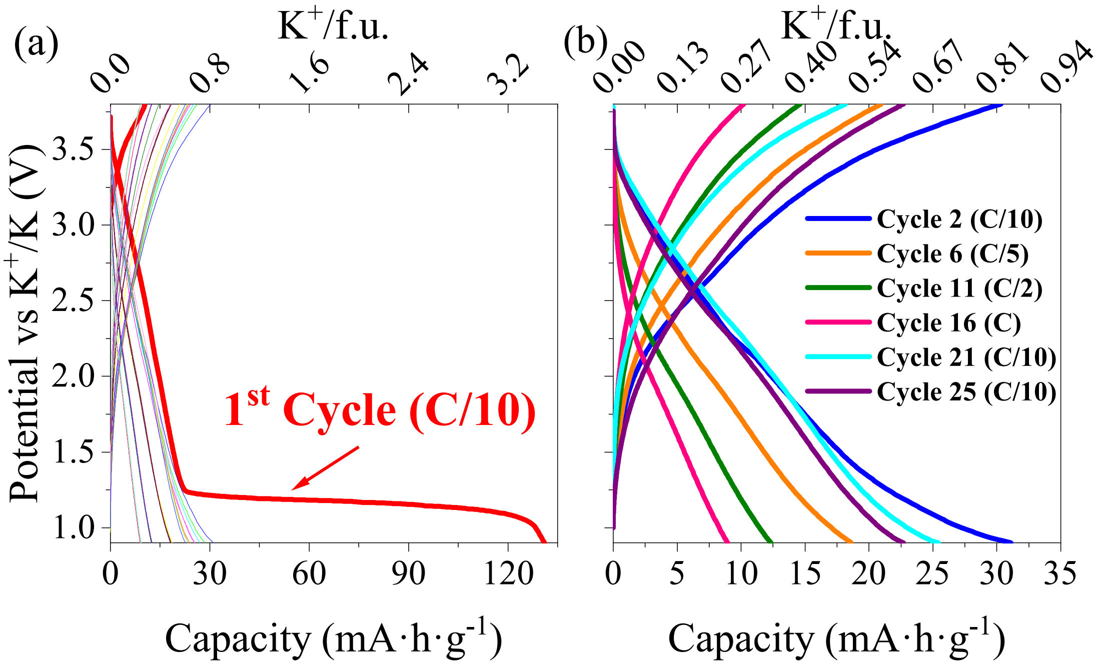

Figure S8. (a) Galvanostatic charge-discharge curves for sample with $K_2V_2Ti_6O_{16}$ nominal composition studied in a potential window between 0.9 – 3.8 V using 3.9 M KFSI (DME) as electrolyte. (b) Enlargement of image (a), without cycle 1.

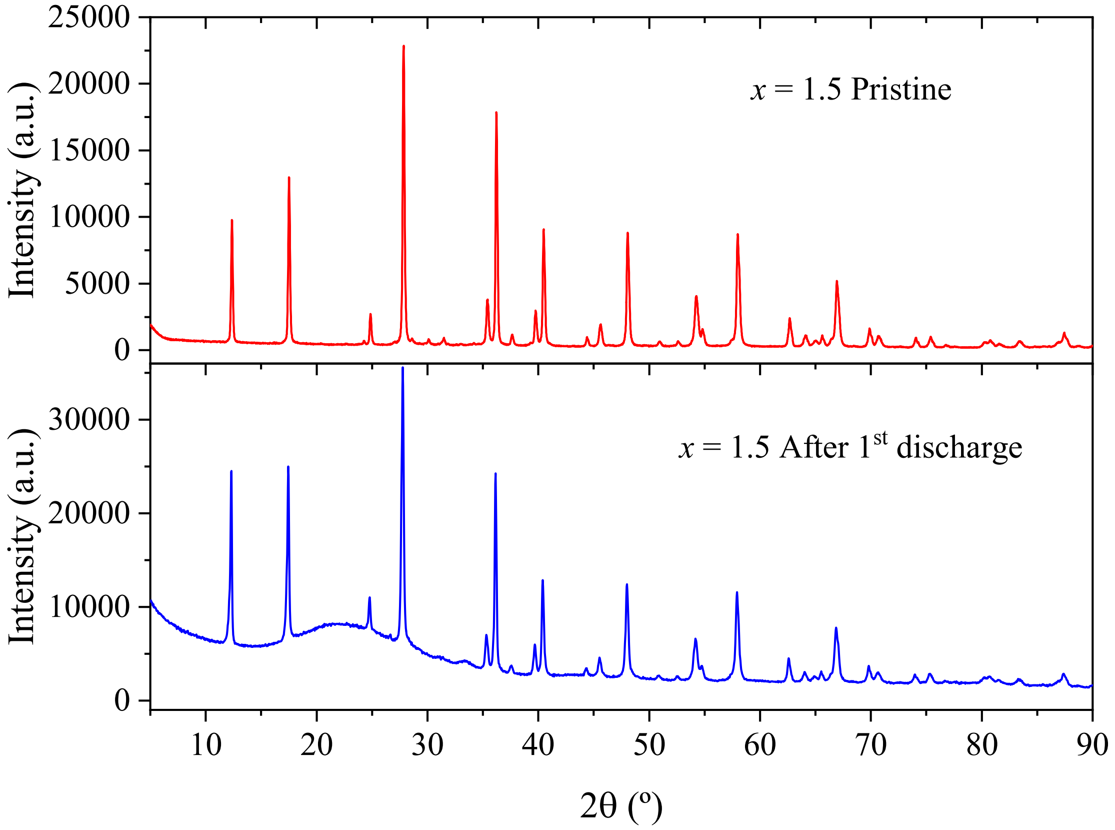

Figure S9. Comparison of the PXRD pattern of the pristine x = 1.5 hollandite and the electrode material after the first discharge and rinsing with TEP. The broad bump centered in the 2θ = 23º region corresponds to the amorphous signal of the glass capillary as well as the conductive carbon.

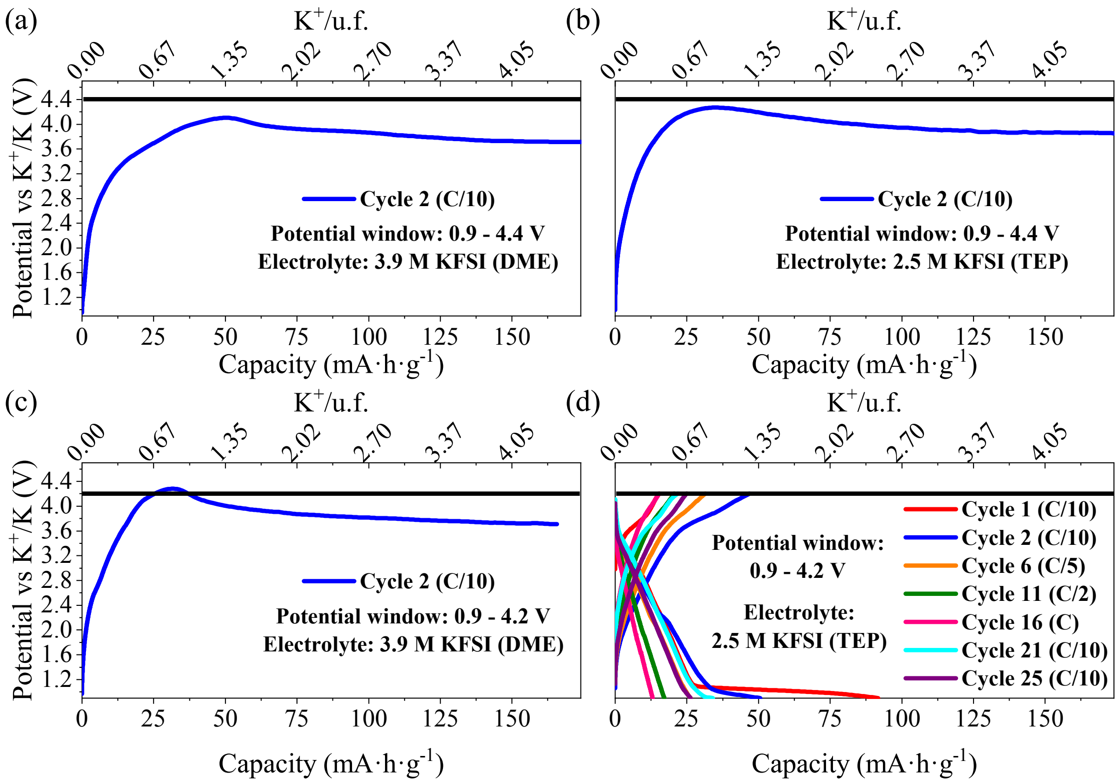

Figure S10. Galvanostatic charge-discharge curves for sample with $K_2V_2Ti_6O_{16}$ nominal composition. (a) Potential window between 0.9 – 4.4 V using 3.9 M KFSI (DME) as electrolyte. (b) Potential window between 0.9 – 4.4 V using 2.5 M KFSI (TEP) as electrolyte. (c) Potential window between 0.9 – 4.2 V using 3.9 M KFSI (DME) as electrolyte. (d) Potential window between 0.9 – 4.2 V using 2.5 M KFSI (TEP) as electrolyte.

As seen in Figure S10, both electrolytes, 3.9 M KFSI (DME) and 2.5 M KFSI (TEP), are unstable up to 4.4 V. However, when the potential window is narrower (0.9 – 4.2 V), it is observed that the 3.9 M KFSI (DME) electrolyte is still not stable up to 4.2 V, it is oxidized above 3.8 V, while the 2.5 M KFSI (TEP) reaches 4.2 V without major decomposition. That is why we use two different potential windows when testing cells using 3.9 M KFSI (DME) or 2.5 M KSFI (TEP).

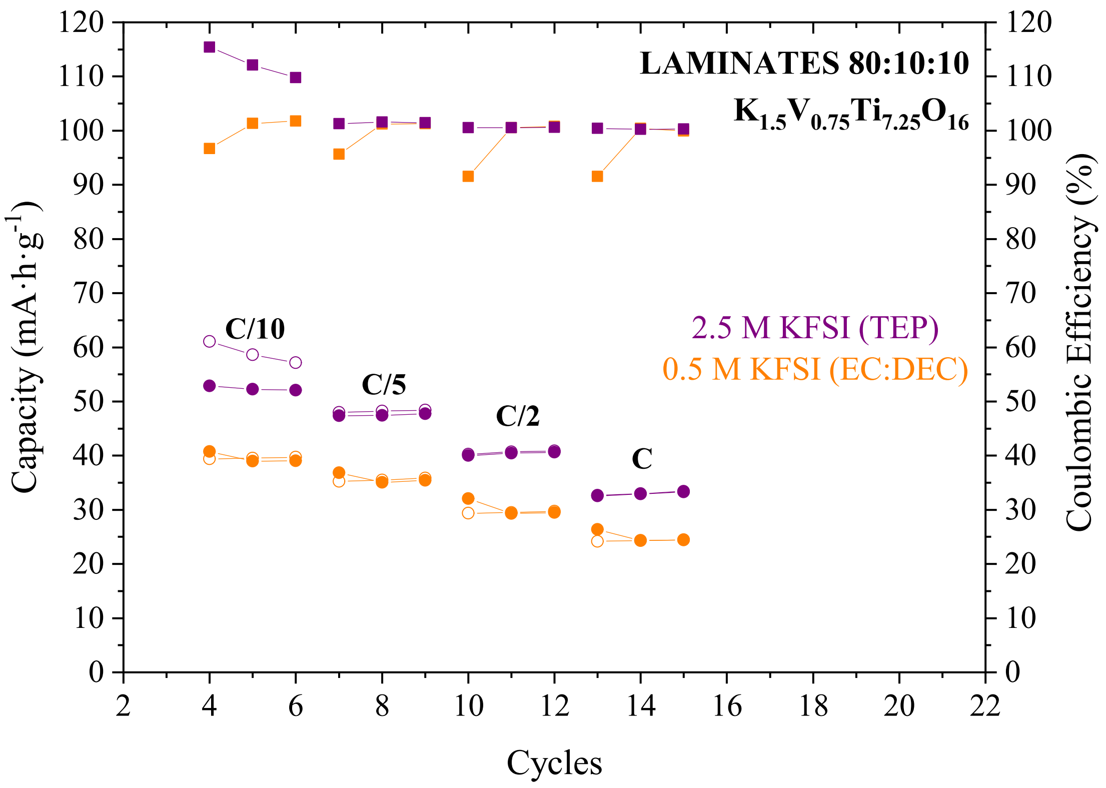

Figure S11. Capacity (circles) and coulombic efficiency (squares) *vs.* cycle number during galvanostatic cycling of $K_{1.5}V_{0.75}Ti_{7.25}O_{16}$. Comparison between two different electrolytes: 2.5 M KFSI (TEP) in purple and 0.5 M KFSI (EC:DEC) in orange.
